# Data availability, reusability, and analytic reproducibility: evaluating the impact of a mandatory open data policy at the journal *Cognition*

**DOI:** 10.1098/rsos.180448

**Published:** 2018-08-15

**Authors:** Tom E. Hardwicke, Maya B. Mathur, Kyle MacDonald, Gustav Nilsonne, George C. Banks, Mallory C. Kidwell, Alicia Hofelich Mohr, Elizabeth Clayton, Erica J. Yoon, Michael Henry Tessler, Richie L. Lenne, Sara Altman, Bria Long, Michael C. Frank

**Affiliations:** 1 Meta-Research Innovation Center at Stanford (METRICS), Stanford University, Palo Alto, CA, USA; 2 Quantitative Sciences Unit, Stanford University, Palo Alto, CA, USA; 3 Department of Psychology, Stanford University, Palo Alto, CA, USA; 4 Harvard Biostatistics, Harvard University, Cambridge, MA, USA; 5 Stress Research Institute, Stockholm University, Stockholm, Sweden; 6 Department of Clinical Neuroscience, Karolinska Institutet, Stockholm, Sweden; 7 Belk College of Business, University of North Carolina at Charlotte, Charlotte, NC, USA; 8 Department of Psychology, University of Utah, Salt Lake City, UT, USA; 9 Liberal Arts Technologies and Innovated Services (LATIS), University of Minnesota, Minneapolis, MN, USA; 10 The Organizational Science Program, University of North Carolina at Charlotte, Charlotte, NC, USA; 11 Department of Psychology, University of Minnesota, Minneapolis, MN, USA

**Keywords:** open data, reproducibility, open science, meta-science, interrupted time series, journal policy

## Abstract

Access to data is a critical feature of an efficient, progressive and ultimately self-correcting scientific ecosystem. But the extent to which in-principle benefits of data sharing are realized in practice is unclear. Crucially, it is largely unknown whether published findings can be reproduced by repeating reported analyses upon shared data (‘analytic reproducibility’). To investigate this, we conducted an observational evaluation of a mandatory open data policy introduced at the journal *Cognition*. Interrupted time-series analyses indicated a substantial post-policy increase in data available statements (104/417, 25% pre-policy to 136/174, 78% post-policy), although not all data appeared reusable (23/104, 22% pre-policy to 85/136, 62%, post-policy). For 35 of the articles determined to have reusable data, we attempted to reproduce 1324 target values. Ultimately, 64 values could not be reproduced within a 10% margin of error. For 22 articles all target values were reproduced, but 11 of these required author assistance. For 13 articles at least one value could not be reproduced despite author assistance. Importantly, there were no clear indications that original conclusions were seriously impacted. Mandatory open data policies can increase the frequency and quality of data sharing. However, suboptimal data curation, unclear analysis specification and reporting errors can impede analytic reproducibility, undermining the utility of data sharing and the credibility of scientific findings.

## 1. Introduction

Access to research data can enable a range of core scientific activities, including verification, discovery, and evidence synthesis. Accordingly, there is growing recognition that data availability is a critical feature of an efficient, progressive, and ultimately self-correcting scientific ecosystem that generates credible findings [1–3]. A minimum level of credibility we would expect of all published findings is that they can be reproduced when the reported analyses are repeated upon the raw data: a concept referred to as computational or analytic reproducibility [4–6]. However, assessment of analytic reproducibility is typically precluded by poor access to research data [7–14]. Furthermore, even when data are shared, inadequate documentation and formatting can render them unusable [10]. Thus, while data sharing has many benefits in principle, the extent to which they are being realized in practice is unclear. Crucially, whether data access enables independent verification of analytic reproducibility is largely unknown.

Any investigation of data-sharing utility faces an immediate impediment: research data are typically not available. The policies of journals and professional societies, such as the American Psychological Association, often fall short of imposing mandatory data-sharing requirements on researchers, and merely recommend that data be ‘available upon request’, if they make any recommendation at all [7,15]. In the absence of stringent community norms or regulations, scientific claims are regularly published without public release of the research data upon which they are based [7,9,14]. Post-publication efforts to obtain data directly from authors frequently go unanswered, or are refused [11–13], and the research community's access to data inevitably gets worse over time since publication as study authors change employment, computer hard drives fail, and e-mail addresses stop working [16]. In a recent attempt [8] to obtain data for some of the most highly cited articles in psychology and psychiatry published over the last decade, the majority of datasets were not made available (76/111, 68%).

The issue of poor data availability in psychology has gained particular urgency in recent years as the credibility of the discipline has come under close scrutiny [17]. A number of journals have responded by introducing ‘open data’ policies which incentivize or mandate data sharing. Initial investigations suggest that some of these policies are associated with a substantial increase in the number of articles reporting that data are publicly available in an online repository [10,18]. For example, in an observational study examining the impact of a voluntary ‘open badges’ scheme at the journal *Psychological Science*, Kidwell *et al*. [10] found that the proportion of articles reporting available data increased from around 2.5% during the pre-policy period to 39.5% by the end of the post-policy period. These trends are certainly encouraging and indicate a healthy response to calls for increased data availability [1–3]. However, simply making data available does not guarantee that the data have utility: Kidwell *et al*. also observed that a substantial proportion (50/111, 45%) of reportedly available data was in fact not available or was incomplete, incorrect, or had insufficient documentation.

Even when data are available, and in-principle reusable, the extent to which they enable analytic reproducibility is uncertain. A smattering of studies have sought to investigate this issue in several scientific disciplines, and most have encountered substantial reproducibility problems [19–21]. For example, Eubank [19] conducted a pre-publication assessment of articles submitted to the *Quarterly Journal of Political Science*, and found that only four out of 24 articles (approx. 17%) were reproducible without author assistance. Similarly, Chang & Li [20] reported that they could only reproduce the key findings of 22 of 59 (approx. 37%) published economics papers (when they had access to study data). On the other hand, Naudet *et al*. [22] recently reported that they could successfully reproduce the primary outcomes of 14 out of 17 (82%) randomized control trials (RCTs) published in *The BMJ* or PLOS Medicine.

In the present investigation, we sought to examine the state of data availability, reusability, and analytic reproducibility within a subfield of psychology. We capitalized on the introduction of a mandatory open data policy at the journal *Cognition* on 1 March 2015 [23], which we anticipated would yield a large corpus of readily available datasets. In study 1, we assessed the policy's impact on data availability, and the extent to which shared data were accessible, complete, and understandable (i.e. in-principle reusable). In study 2, we conducted an in-depth assessment of analytic reproducibility for a subset of outcomes reported in a sample of articles with in-principle reusable data.

## 2. Study 1

In study 1, we conducted a retrospective pre-/post-assessment of *Cognition'*s open data policy. The policy required that authors make relevant research data publicly available prior to publication of an article. We began by examining how the policy impacted the frequency of data available statements using an interrupted time-series analysis to control for secular trends. We then assessed whether purportedly available data were in-principle reusable; specifically, were data: (i) accessible; (ii) complete; and (iii) understandable? We hypothesized that the stringent nature of a mandatory policy would lead to a substantial increase in the number of data available statements, but anticipated shortcomings in data reusability based on previous observations in [10].

### 2.1. Methods

The study protocol (hypotheses, methods, and analysis plan) was preregistered (https://osf.io/q4qy3/). All deviations from this protocol or additional exploratory analyses are explicitly acknowledged.

#### 2.1.1. Sample

The sampling frame included all 591 empirical articles^1^ published in the journal *Cognition* from March 2014 to March 2017, inclusive. Non-empirical articles such as reviews, meta-analyses, simulations, commentaries or editorials were not considered. Articles are referred to here using a unique five-character ID code (e.g. QrKHp). A bibliography file is available where ID codes can be matched to article references (https://osf.io/pnvf8/).

An article's submission date determined whether the open data policy applied. There were 417 articles in the pre-policy period and 174 articles in the post-policy period. The earliest submission date was 2 December 2009 (1915 days pre-policy) and the last submission date was 5 August 2016 (523 days post-policy).

#### 2.1.2. Design

This was a quasi-experimental pre-/post-design in which individual sampling units (articles) had previously been exposed to a manipulation (a new open data policy) determined by factors outside our control.

##### 2.1.2.1. Manipulated variable

A mandatory open data policy was introduced at the journal *Cognition* on 1 March 2015 [23] and applied to all articles *submitted*^2^ on or after this date (S. Sloman 22 January 2017, personal communication). The policy required that prior to publication authors made their data available in the article's electronic supplementary material section, or a suitable third party repository. Additionally, the policy required that data should be shared in a form that enables reuse and analytic reproducibility [24].

We are not aware of any pertinent co-interventions occurring during the assessment period; however, the introduction of the policy did coincide with the arrival of a new Editor-in-Chief [23]. Also note that, although the editorial announcing the policy was formally published on the date that the policy came into effect (1 March 2015), it was also available online from 20 November 2014, which could have led to anticipatory effects (see 2.3 Discussion). The content of the new policy has remained unaltered for the duration of the assessment period (S. Sloman 26 January 2017, personal communication). There was no explicit open data policy prior to the new policy (M. Tsakiris 5 March 2018, personal communication).

##### 2.1.2.2. Measured variables

For each article, data extractors (coders) recorded the nature of the data availability statement (data available statement, data not available statement, no statement), reasons provided (if any) for lack of data availability, type of accessibility (available upon request, personal website, *Cognition* supplementary information, third-party repository), actual accessibility (i.e. could file(s) be successfully downloaded and opened: yes, no, some files only), file format (.txt, .dat, .csv, .xls or .xlsx, .doc or .docx, .sav, .dta, unclear, other), completeness (all data appear available, only some data appear to be available, unclear), understandability (yes, partly, no), analysis scripts (present/absent) and inclusion of a licence (CC-BY, CC-0, other, no licence). For some analyses, accessibility, completeness, and understandability were considered as a composite measure of ‘in-principle reusability’.

#### 2.1.3. Procedure

For each article, coders extracted information related to the measured variables (above) and entered it into a Google Form (https://osf.io/qr6e4/) that employed a multiple choice format in order to standardize responses. If the article had a data available statement, coders were asked additional questions about in-principle reusability. Specifically, ‘Were you able to successfully download and open the data file?’ (accessibility), ‘Do all of the data needed for evaluation and reproduction of the research appear to be available after brief review?’ (completeness), and ‘Are the data understandable after brief review?’ (understandability). Data files were considered incomplete if they did not contain ‘raw’ data (i.e. at a minimum, individual participant-level data) and/or data for all measured variables reported in the article. To determine ‘understandability’, coders were instructed to check for clear labelling in the data file itself and evaluate any additional documentation (such as a ‘codebook’ or ‘data dictionary’) that was provided alongside the data files.

All team members were either currently engaged in or had completed graduate-level training in a scientific discipline, typically psychology. All five coders successfully completed a training phase in which their coding of five preselected training articles reached more than 90% agreement with a ‘gold standard’ established by a single author (T.E.H.). All team members were required to notify T.E.H. if they were assigned an article with which they had a conflict of interest (e.g. they were an author), but this did not occur.

Coders were not blind to the study hypotheses; however, the data extraction protocol mostly left little room for subjective interpretation (see https://osf.io/qr6e4/). Articles were randomly selected and assigned to coders in order to ameliorate selection bias and ensure that any coder drift would affect pre-policy and post-policy articles evenly. Coders could request batches of up to 20 articles at a time. The articles were single coded and ultimately distributed among the five coders in the following quantities: 115, 74, 176, 173 and 53.

### 2.2. Results

Ninety-five per cent confidence intervals (CIs) are based on the Wilson method with continuity correction for binomial proportions [25] and the Sison–Glaz method for multinomial proportions [26].

#### 2.2.1. Data availability statements

For each article, coders recorded whether there was a ‘data available’ statement, ‘data not available’ statement or no statement related to data availability. We did not encounter any ‘data not available’ statements and the editorial team confirmed that they were not aware of any case during the assessment period when authors requested exemption from the policy (S. Sloman 22 January 2017, personal communication). Consequently, we focused our analysis on the proportion of data available statements (DAS; see second row of figure 2). We observed a considerable proportion increase in DAS inclusion between the pre-policy (104/417 = 25%, CI [21, 29]) and post-policy period (136/174 = 78%, CI [71, 84]). A Pearson's chi-squared test of independence was statistically significant (*χ*^2^(1, *n* = 591) = 144.18, *p* < 0.001).

**Figure 1.**
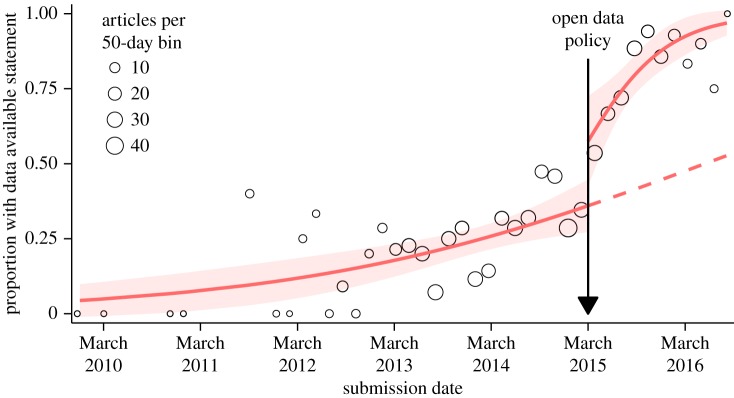
Proportion of articles with data available statements as a function of submission date across the assessment period. For ease of presentation, circles indicate proportions in 50-day bins with the circle area representing the total number of articles in each bin (but note that the analysis model was fitted to individual articles). Solid red lines represent predictions of an interrupted time-series analysis segmented by pre-policy and post-policy periods. The dashed red line estimates, based on the pre-policy period, the trajectory of data available statement inclusion if the policy had no effect. The model is linear on the logit scale, whereas the *y*-axis of the figure is on the probability scale, which is a nonlinear transformation of the logit. Confidence bands (red) indicate 95% CIs. Note that the small article numbers in the extremes of the graph are due to long submission-to-publication lag times. Our sample selection was based on the publication date, but it is the submission date which determines whether an article falls within the pre-policy or post-policy period.

To estimate the causal effect of the policy independent of any contemporary secular (long-term) trends in DAS inclusion, we conducted an interrupted time-series analysis^3^ (figure 1). This approach involves modelling separate trajectories over time for DAS inclusion in the pre-policy versus the post-policy period, allowing for a discontinuity at the moment of policy implementation [27]. We can therefore estimate the causal effect of the policy at the moment it was implemented, as well as its effect on a possible existing secular trend. The causal interpretation relies on the assumption that articles submitted immediately before and immediately after the policy implementation were similar [28]. This requires, for example, that there were no other interventions concurrent with the new policy that affected DAS inclusion, and that authors did not preferentially submit their articles just prior to policy implementation to avoid having to release their data.^4^

We conducted the interrupted time-series analysis by fitting a logistic regression model in which the units of analysis were articles. We regressed an indicator for whether the article included a DAS on an indicator for whether an article was published after versus before the policy (‘post’), a linear effect of time since policy implementation in units of 50 days (‘time’),^5^ and their interaction:

$$\text{logit(available)}=\beta_{0}+\beta_{1}\;\text{time}+\beta_{2}\;\text{post}+\beta_{3}\;\text{time}*\;\text{post}.$$

This model is equivalent to the following pair of models for the pre-policy period and the post-policy period separately:

$$\begin{array}{rl} & \text{logit}(\text{available}\;|\;\;\text{post}=0)=\beta_{0}+\beta_{1}\text{time}\\ & \text{logit}(\text{available}\;|\;\;\text{post}=1)=(\beta_{0}+\beta_{2})+(\beta_{1}+\beta_{3})\text{time.} \end{array}$$

Thus, exp(*β*_2_) represents the odds ratio for DAS inclusion comparing an article published immediately after policy implementation with an article published immediately before. exp(*β*_1_) represents the odds ratio of DAS inclusion comparing an article published at any given time in the pre-policy period with an article published 50 days prior, and exp(*β*_3_) is the multiplicative factor by which this odds ratio increased in the post-policy period. For interpretability, we present all subsequent results on the risk ratio scale using Zhang & Yu's [29] conversion.^6^

During the pre-policy period, we observed a baseline secular trend towards increasing rates of DAS inclusion. Specifically, each 50-day passage of time in the pre-period was associated with an estimated 1.04-fold (CI [1.02, 1.06], *p* = 0.002) increase in the probability of DAS inclusion. After the open data policy was introduced, there was a substantial ‘level change’ such that an article published immediately after the policy had an estimated 1.53-fold higher probability (CI [1.10, 1.90], *p* = 0.017) of DAS inclusion than an article published immediately before the policy. Furthermore, the trend over time towards increasing rates of DAS inclusion appeared to accelerate in the post-period; the secular trend in the post-period was an estimated 1.14-fold (CI [1.04, 1.26], *p* = 0.010) greater than that in the pre-period. As an alternative interpretation, we estimate that 5% (CI [0, 10], *p* = 0.022) of the 50-day secular trend towards increasing DAS inclusion in the post-period reflects the baseline secular trend alone, that 65% (CI [40, 90], *p* < 0.001) reflects the effect of the policy alone and that 30% (CI [7, 53], *p* < 0.001) reflects acceleration in the baseline secular trend due to the policy—that is, the interaction of the policy with the secular trend [30–32]. In this last analysis, we approximated standard errors using the delta method and VanderWeele's [33] approximate one-to-one transformation between the odds ratio and the risk ratio. *p*-values represent one-sided tests of the null hypothesis that each proportion is truly zero.

According to the availability statements, data for two articles (1%) were obtainable via a personal webpage, data for 27 articles (11%) were obtainable via a third party repository and data for 208 articles (87%) were obtainable via the online supplementary materials hosted alongside the article by *Cognition* (the default option, see electronic supplementary material A). A final three articles (1%) indicated data were available via other means: in another article (*n* = 2) or in a table in the current article (*n* = 1). No articles indicated that data were available upon request from the authors.

#### 2.2.2. In-principle data reusability

**Figure 2.**
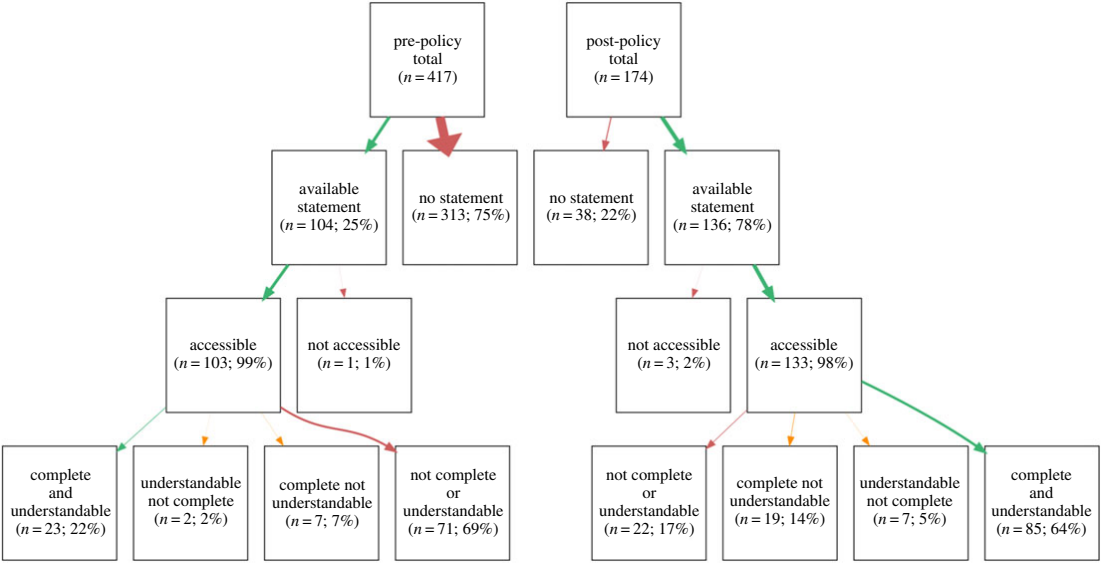
Counts and percentages for articles in the pre- and post-policy periods with available statements, accessible data, complete data and understandable data. Only accessible, complete and understandable data are considered ‘reusable in principle’. Arrow size represents the proportion of total articles.

When articles had data available statements, we also assessed whether the data were in-principle reusable: specifically, were the data accessible, complete and understandable?^7^ We were able to successfully download and open data files (accessibility) in all but four cases (see third row of figure 2). The proportion of datasets with complete and/or understandable data is indicated in the fourth row of figure 2.

Overall, the pre-policy period had 23 in-principle reusable datasets (22% of the 104 articles with data available statements during this period; 6% of all 417 articles assessed during this period). By contrast, the post-policy period had 85 in-principle reusable datasets (62% of the 136 articles with data available statements during this period; 49% of all 174 articles assessed during this period).

Exploratory analyses (not preregistered) indicated a significant improvement in in-principle reusable between the pre-policy and post-policy period. A Pearson's chi-squared test of independence was statistically significant (*χ*^2^(1, *n* = 591) = 154.38, *p* < 0.001). An interrupted time-series analysis indicated a baseline secular trend towards increasing rates of in-principle reusable and a significant ‘level change’ towards higher in-principle reusable post-policy (for details, see electronic supplementary material C).

Eighteen articles included analysis scripts. We did not evaluate these in detail; however, only eight of the scripts were shared alongside data that were deemed reusable. We also recorded information about file formats and licensing, which is presented in electronic supplementary material B.

### 2.3. Discussion

In study 1, we observed a substantial increase in data available statements and in-principle reusability following the introduction of a mandatory open data policy at the journal *Cognition*. However, compliance with the policy was not ideal: in the post-policy period 22% of articles did not contain data available statements and 38% of reportedly available datasets did not appear to be reusable in principle. Nevertheless, towards the end of the assessment period the situation was generally improving: the proportion of data available statements was approaching 100% and in-principle reusability was approximately 75%.

To what extent can the observed changes in data availability and reusability be causally attributed to the policy intervention? A causal interpretation faces several threats to validity due to the inherent limitations of the study's non-randomized design. For example, one might postulate that the increase in data availability and reusability was due to growing awareness of open science issues in the population of authors submitting to *Cognition* (a ‘maturation’ threat), or events occurring during the evaluated time period, such as the publication of a landmark paper on replicability [34]. Interestingly, we did observe an increase in data available statements and in-principle reusability prior to the introduction of the policy, perhaps reflecting growing awareness in the field of psychology about the importance of transparency in general and data sharing more specifically [2,35]. The online publication of the editorial announcing the open data policy four months prior to policy implementation may also have led to some anticipatory effects [23]. We attempted to minimize these threats to validity by employing an interrupted time-series analysis to estimate the causal effect of the policy independent of any contemporary secular trends [27]. The analysis indicated an increase in data availability and reusability above and beyond what one might have expected if the observed secular trend had continued. Additionally, we are not aware of any concurrent event(s) that could explain the abrupt changes observed at the specific time of policy introduction, although it remains possible that such an event(s) occurred. An additional source of bias worth noting is that coders were not blind to the study hypotheses. However, because the data extraction protocol mostly left little room for subjective interpretation, we consider the potential for bias to be low.

Straightforward causal conclusions are also complicated by the possibility of self-selection bias. For example, it could be that researchers positively inclined towards open science were attracted to the journal by the new policy, and other researchers deterred. If there were such a ‘population shift’, the observed increases in data availability and in-principle reusability may overestimate the efficacy of the policy for researchers who are not already inclined to adopt transparent research practices. It is not straightforward to ascertain definitively whether such a population shift occurred. Nevertheless, we performed two exploratory analyses which partially address the population shift hypothesis (presented in detail in electronic supplementary material D). Firstly, we investigated whether there was a persistent population of authors who regularly publish in *Cognition*, and then examined if these authors continued to publish there after the new open data policy was introduced. We calculated a yearly ‘author retention index’ which represented the overlap between authors publishing at the journal in a given year relative to the previous 3 years. The author retention index was stable both before and after the policy intervention (see electronic supplementary material, figure D1). Thus, we observed no evidence that this group of authors were deterred by the policy. However, arguably these authors are also the least likely to abandon the journal. Authors who were less committed to the journal may have been more inclined to submit elsewhere if they disliked the policy. Secondly, we examined whether there was a fall in journal productivity (number of publications per year) after introduction of the policy. This would be consistent with a large number of authors being deterred from submitting their work to *Cognition* (although other explanations are also possible). In fact, journal productivity appears to have increased from before to after policy introduction (see electronic supplementary material, figure D2). This appears inconsistent with the notion that large numbers of authors were deterred by the new open data policy. However, it is also possible that the number of authors attracted by the policy was larger than the number who were deterred. Overall, these exploratory analyses found no evidence that the observed changes in data availability and in-principle reusability were due to a population shift, but they do not rule out that such a shift occurred.

Our assessment revealed that several aspects of the open data policy could be improved upon. Most obviously, the occurrence of articles in the post-policy period without available data, and with data that did not appear reusable in principle, suggests that the policy was not being consistently enforced. A major contributor to poor in-principle reusability could be a lack of knowledge on behalf of authors. Providing clear guidelines about best practices in data stewardship (structure, documentation, etc.) may help to improve standards [36]. Time is of course a scarce resource, and the ability of editors and/or reviewers to assess data availability and reusability may be limited. Providing editors with a clearly structured checklist might improve the efficiency and consistency of assessment. Additionally, the task of data assessment could be assigned to a specific member of the assistant editorial staff. The amount of time allocated to checking policy compliance will require a weighing of potential costs and benefits that will vary between individuals and, in this case, journals. We believe that high-quality data sharing is an essential feature of the scientific endeavour [1,2] and warrants additional resource investment.

Several less crucial, but nevertheless important, aspects of the current data-sharing policy could also be improved. Firstly, many articles were accompanied by a link to ‘supplementary data’ files that, upon closer inspection, contained research materials rather than data. This is reflected in the large number of .doc and .pdf file formats we encountered when trying to locate data (electronic supplementary material B). Clearer labelling would help to avoid giving the impression that data were available when in fact they were not. Secondly, approximately half of the data we encountered were accompanied by a licence (typically CC-BY) and half were not. More consistent licensing would reduce uncertainty about the acceptable conditions of data reuse [37]. Thirdly, by far the most popular option for data hosting was *Cognition*'s online supplementary materials section. While this might seem an obvious default, there is some evidence that such systems cannot be relied upon as ‘broken links’ proliferate over time, rendering research resources unavailable [38]. To ensure the persistence of data (and other materials), it might be preferable to recommend use of a dedicated third-party data repository where files can be registered (time-stamped and made read-only), and issued digital object identifiers (DOIs).

In conclusion, the introduction of an open data policy at *Cognition* had a substantial positive impact on data availability and in-principle reusability. However, more stringent guidelines and enforcement will probably be required to maintain high availability rates and maximize the utility of data sharing. Finally, it is likely that our relatively brief assessment of in-principle reusability underestimates actual reusability in practice. Being able to verify the analytic reproducibility of reported outcomes is necessary to establish a baseline standard for all subsequent data reuse, and is a minimal threshold of scientific credibility [37]. We explore this issue in study 2.

## 3. Study 2

In study 2, for a subset of articles that had in-principle reusable data, we assessed the analytic reproducibility of a set of outcomes related to a substantive finding. When we encountered problems reproducing target outcomes, we contacted the original authors to ask for assistance. Based on previous investigations [19–21] and given the absence of a policy requirement to share analysis scripts, we anticipated some difficulties verifying the analytic reproducibility of reported findings.

### 3.1. Methods

The study protocol (hypotheses, methods and analysis plan) was preregistered (https://osf.io/q4qy3/). All deviations from this protocol or additional exploratory analyses are explicitly acknowledged.

#### 3.1.1. Sample

We assessed the analytic reproducibility of a subset of descriptive and inferential statistics reported in 35 of the articles already determined to have in-principle reusable data in study 1. Further details of the selection process are outlined in detail below.

A ‘triage pool’ was populated incrementally as study 1 coders identified articles that had available and in-principle reusable data (accessible, complete and understandable). Articles in the triage pool were randomly selected and examined for suitability by a single author (T.E.H.). Only articles reporting at least some behavioural data and quantitative analyses were considered. Within each article, a single author (T.E.H.) identified a subset of ‘relatively straightforward and substantive’ outcomes to be targeted by the reproducibility check.

Inevitably, there was an element of subjectivity to this process; however, the following guidelines were adopted. (i) A ‘relatively straightforward’ analysis was one which would probably feature in an introductory-level psychology statistics textbook [39] and could therefore be comfortably performed by a competent graduate-level (or higher) psychology student or researcher. Examples include correlations, *t*-tests and ANOVAs. (ii) A ‘substantive’ finding was one which was emphasized in the article's abstract, a figure or table. For a given article, the target outcomes were a coherent set of inter-related values (descriptive and inferential statistics) that supported the identified substantive finding. As such, we attempted to reproduce a range of different values in each reproducibility check, including means, medians, counts, standard deviations, confidence intervals, effect sizes, degrees of freedom, test statistics, and *p*-values. The first set of coherent analyses encountered in the article that met the above criteria were chosen as the target outcomes. This usually corresponded to one or two paragraphs in the results section of a single experiment. Typically, there were many other findings reported in a given article that we did not attempt to reproduce.

A sample size was not preregistered: we continued running reproducibility checks on an *ad hoc* basis until we reached the limits of our personnel resources. Ultimately 108 articles had in-principle reusable data and entered the triage pool (see study 1). Forty-seven of these articles were assessed for eligibility, 12 of which were rejected because a finding that was both ‘straightforward’ and ‘substantive’ could not be readily identified (according to the above criteria). Thirty-five articles were deemed eligible for reproducibility checks, of which five were from the pre-policy period and 30 were from the post-policy period. A bibliography file is available where article ID codes can be matched to their references (https://osf.io/9a6qu/).

#### 3.1.2. Design

This was a non-comparative case-study design.

##### 3.1.2.1. Measured variables

For analytic reproducibility checks, we recorded outcomes at multiple levels of granularity as outlined below.

*Classification of reproducibility errors*. We adopted an error classification scheme to capture four types of reproducibility problem. An *insufficient information error* occurred when the original analysis specification was ambiguous or absent to such an extent that we could not proceed with the reanalysis. When we could attempt a reanalysis, any numerical differences between original (reported) outcomes and reanalysis (obtained) outcomes were quantified in terms of percentage error (PE):

$$\text{PE}=\frac{\left|\text{obtained}-\text{reported}\right|}{\text{reported}}\times 100.$$

We used PE^8^ to define whether there was a *minor numerical error* (a PE greater than 0% but less than 10%) or a *major numerical error* (a PE greater than or equal to 10%). Given their predominant role in statistical inference [40], *p*-values were treated as a special case: if the reported *p* fell on the opposite side of the 0.05 boundary^9^ relative to the obtained *p,* this was classified as a *decision error*.

*Classification of article-level reproducibility outcomes*. After tallying up the four types of errors (above), we determined an overall reproducibility outcome for each case (article target outcomes) based on the following. If there were *any* insufficient information errors, major numerical errors or decision errors remaining after author assistance, then the case was considered not fully reproducible. If there were only minor numerical errors, or no discrepancies, then the case was considered reproducible. We also considered whether author assistance was required to resolve any errors. Consequently, each case was assigned to one of the following categories: reproducible, reproducible with author assistance only, not fully reproducible, or not fully reproducible despite author assistance.^10^

*Implications of non-reproducibility for original conclusions*. An important question is whether any non-reproducibility of target outcomes affected the corresponding substantive conclusions presented in the original articles. This is a complex issue to address and cannot be straightforwardly condensed into a single quantitative index. Instead, it is necessary to carefully consider multiple factors, such as the presence/absence of decision errors, the number of target outcomes that could not be reproduced, the type of outcomes that could be reproduced, the difference in magnitude of effect sizes, and the predictions of the specific hypothesis under scrutiny. Consequently, we conducted a qualitative assessment of the pattern of reproducibility issues affecting each article, and determined on a case-by-case basis whether there were likely to be substantial implications for the original conclusions. These assessments were not based on preregistered criteria, and are necessarily subjective. We have provided a rationale supporting each determination in the series of reproducibility vignettes presented in electronic supplementary material E.

*Causal locus of non-reproducibility*. Where possible, we attempted to identify the causal locus of non-reproducibility within the analysis pipeline. Detailed information about the nature of the reproducibility issues and possible causes is contained in the individual ‘reproducibility reports’ (available here: https://osf.io/p7vkj/) we wrote for each reproducibility check (see Procedure below). However, in order to more succinctly convey this dense body of information, we also recorded the number of discrete reproducibility issues we encountered according to the following *post hoc* (not preregistered) classification scheme: typographical issue (i.e. a ‘typo’ probably due to the inaccurate transfer of information from the analysis output to the manuscript), inadequate specification of analyses (i.e. it was not clear from the article how to reproduce the analyses), original analysis issue (a problem related to the authors' original analyses), data file issue (a problem related to the shared data file) or unidentified cause. Examples of these issues can be found in the results section. We also recorded whether such issues were resolved by author assistance.

Note that there is not necessarily a direct mapping between discrete reproducibility issues and the number of reproducibility errors because several errors could be attributable to a single cause. For example, misspecification of an ANOVA could lead to reproducibility errors for all outcomes associated with that ANOVA. The locus of reproducibility errors was often identified based on information provided by the original authors; however, all classifications were made independently by our research team.

#### 3.1.3. Procedure

At least two members of our team attempted to reproduce the target outcomes for each article by repeating the reported analysis. When any reproducibility issues were encountered, we attempted to resolve them through contact with the original authors. Further procedural details are outlined below.

To minimize errors, we employed an analysis co-piloting model [41] in which every reproducibility check involved the input of at least two team members. All pilots/co-pilots had experience conducting the types of data analyses typically found in an introductory psychology statistics textbook [39], and using R Markdown for writing reproducible analysis reports. The initial reproducibility check was conducted independently by the ‘pilot’, who was subsequently accompanied by at least one other team member acting as ‘co-pilot’. The co-pilot's job was to verify the analyses of the pilot and work with them to resolve any outstanding issues. The pilot and co-pilot prepared a joint report detailing the analyses and outcomes of the reproducibility check (available here: https://osf.io/p7vkj/). These reports were written in a ‘literate programming’ style (regular prose interleaved with analysis code) using a custom R Markdown template in order to facilitate reproducibility of our own analyses.

The sole aim of the reproducibility checks was to reproduce the specified target outcomes using the available data files, information provided in the original article and any other additional documentation (e.g. codebook or analysis scripts). It was not our goal to attempt alternative analyses. We were only interested in analytic reproducibility for the purposes of the current investigation.

When pilots encountered ambiguous or absent analysis specifications, they were encouraged to note an ‘insufficient information error’ rather than engage in lengthy guesswork. This approach reflects that we were interested in the extent to which the article's findings could be reproduced using the original authors' analysis specification. The outcome of any guesswork on the part of the pilot may be more reflective of their idiosyncratic detective skills than the reproducibility of the target outcomes. In addition, such guesswork could be unbounded and ultimately prove fruitless. Nevertheless, some guesswork inevitably became part of the reanalysis process when it seemed straightforward to run a small number of plausible analyses. For example, running a chi-squared test with and without continuity correction, or a Welch *t*-test instead of a Student *t*-test, to see if either of the outputs matched the reported value.

### 3.2. Results

We present the results of our reproducibility assessments at several layers of granularity. The most detail is contained in each assessment's R Markdown report (https://osf.io/p7vkj/), which outlines the reanalysis process step by step through interleaved prose and analysis code. Electronic supplementary material E contains a more condensed summary of each report in a series of ‘reproducibility vignettes'. Below, we present a quantitative assessment of our findings, at both the article level and outcome level, accompanied by illustrative examples where relevant.

We initially encountered errors in 24 of the 35 reproducibility assessments (69%, CI [51, 83]). However, in all of these cases, we requested and received assistance from the original authors, which resolved many issues. Ultimately, the target outcomes in 11 (31%, 95% CI [17, 51]) articles were reproducible without any author assistance, target outcomes in 11 (31%, 95% CI [17, 51]) articles were reproducible with only author assistance and 13 (37%, 95% CI [23, 57]) articles contained some outcomes that were not reproducible despite author assistance.^11^

In total, we attempted to reproduce 1324 individual values and, after author assistance was provided, 64 major numerical errors (5%, CI [4, 6]) and two insufficient information errors remained. There were no decision errors. There were also 146 minor numerical errors which we did not think warranted further consideration because of their small magnitude.

Importantly, in almost all cases where target outcomes were not fully reproducible (*n* = 10), it appeared unlikely that the reproducibility issues we encountered have substantial implications for the corresponding conclusions outlined in the original articles. A detailed rationale for this determination is provided on a case-by-case basis in electronic supplementary material E. In summary, there were no decision errors for any cases, and in most cases reproducibility issues were isolated to a relatively small number of major errors. Sometimes major errors were constrained to values that appeared relatively inconsequential for the overall findings, such as degrees of freedom. There were major errors related to effect sizes in only five cases, and for four of these the magnitude of the error was low (COGGV, DvcDF, jjmld, bPJii). In one case (OvGDB), there was a larger discrepancy between the effect sizes (reported *d* = 0.65 versus reanalysis *d* = 0.23) but all other target outcomes (including other effect sizes and inferential statistics) were successfully reproduced, and the reanalysis effect size still appears consistent with the hypothesis under scrutiny. Note that there were three additional ‘unclear’ cases where we did not have sufficient information to proceed with an aspect of the reproducibility assessment and could therefore not make an informed judgement about the validity of the original conclusions.

**Figure 3.**
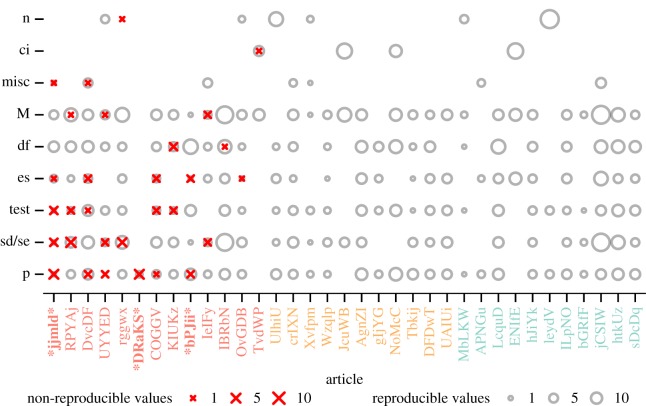
All 1324 values were checked for reproducibility as a function of article and value type (n = count/proportion; ci = confidence interval; misc = miscellaneous; M = mean/median; df = degrees of freedom; es = effect size; test = test statistic; p = *p*-value; sd/se = standard deviation/standard error. Bold red **X** marks indicate non-reproducible values (major errors) and grey circles indicate reproducible values. Symbol size represents the number of values. Both axes are ordered by an increasing number of errors towards the graph origin. The article colours represent the overall outcome: not fully reproducible despite author assistance (red), reproducible with author assistance (orange) and reproducible without author assistance (green). For articles marked within asterisks (*), the analysis could not be completed and there was insufficient information to determine whether original conclusions were affected. In all other cases, it is unlikely that original conclusions were affected.

The errors broken down by outcome type are displayed in figure 3. Of the major errors, 17 (27%) were standard deviations or standard errors, 17 (27%) were *p*-values, 10 (16%) were test statistics, such as *t*- and *F*-values, eight (12%) were effect sizes such as Cohen's *d* or Pearson's *r* values, four (6%) were means or medians, four (6%) were degrees of freedom, one (2%) was a count or proportion, one (2%) was a confidence interval and two (3%) were other miscellaneous values.

**Figure 4.**
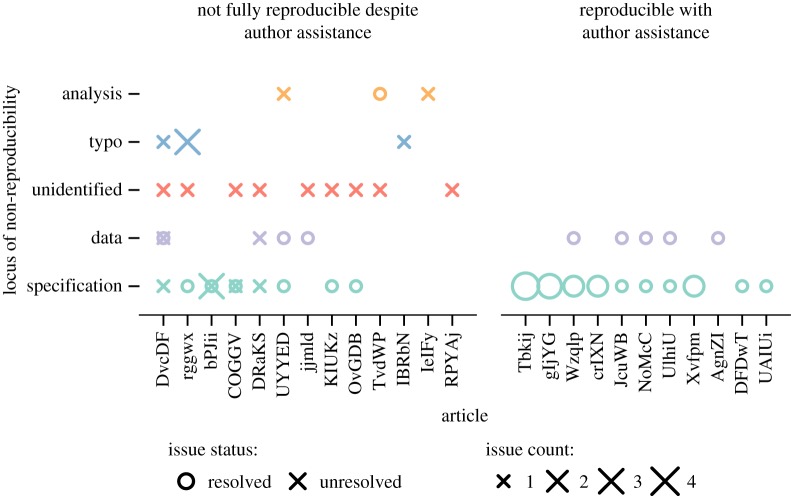
Locus of non-reproducibility based on discrete issues identified in each article. Circles indicate reproducibility issues resolved through author assistance, and X marks indicate unresolved reproducibility issues. Symbol size represents the number of discrete reproducibility issues. Left panel represents articles that were not fully reproducible despite author assistance (some issues may have been resolved but others remain). Right panel represents articles that were reproducible with author assistance (all issues were resolved). Both axes are ordered by an increasing number of discrete reproducibility issues towards the origin.

It was not always possible to definitively isolate the causal locus of non-reproducibility; however, we have identified some likely culprits in most cases. The examples provided below are illustrative rather than comprehensive and readers should consult electronic supplementary material E for details. For each reproducibility check, we labelled discrete issues that had caused reproducibility problems according to a *post hoc* classification scheme (figure 4). In total, we isolated 57 reproducibility issues which we mapped to four categories. Of these 57 issues, there were 30 (53%) issues related to incomplete or ambiguous specification of the original analysis procedure. For example, data exclusions reported in the article could not be mapped to the corresponding values in the data file (Tbkij), ANOVA corrections for violations of sphericity not reported (gIjYG), inferential tests not identified (Wzqlp), non-standard methods for calculating standard errors not reported (crIXN), pre-analysis aggregation steps not reported (UAIUi), and incomplete specification of a linear mixed model (NoMcC). Ten (18%) issues were related to missing or incorrect information in the data file;^12^ for example, data files containing rounded data, rather than raw data (Wzqlp), relevant variables not included (DRaKS), data entry errors (DvcDF), and errors introduced through manual editing of the file (UYYED). Five (9%) issues were typographical in nature and appeared to be caused by human error during the transfer of information between analysis output and the research manuscript. Three (5%) issues were related to errors in the original authors' analyses, problems that were often detected when original authors attempted to reproduce their own findings following our contact. For example, values reported in the paper not from the most up-to-date version of the analyses (UYYED), and confidence intervals calculated based on a formula that contained an error (TvdWP). Finally, the locus of nine (16%) reproducibility errors could not be identified, even after correspondence with the original authors.

Out of the 57 discrete reproducibility issues, 33 (58%) were resolved through author assistance. Of these, 24 (73%) issues were resolved by clarifying ambiguous or incomplete analysis specifications. Eight (24%) issues were resolved by replacement of incomplete or corrupted data files. One (3%) issue was resolved when provision of the authors' code enabled us to identify an error in the original analysis.

We did not record the precise time it took to complete the reproducibility checks. However, we estimate that assessments in the ‘reproducible’ category took 2–4 person hours, and assessments in the ‘reproducible with author assistance’ and ‘not fully reproducible, despite author assistance’ categories took 5–25 person hours. The overall completion time for assessments that involved author assistance was typically between a few weeks and several months due to back-and-forth communications between pilots, co-pilots, and authors. We only consulted authors after extensive efforts by at least two team members to reproduce the target outcomes. There were no cases where we retrospectively felt that author contact was unjustified because we had missed relevant information in the article.

In cases of non-reproducibility, we did not ask authors to take any additional follow-up actions. However, in three cases the authors indicated to us that they would publish a formal correction. To our knowledge, one correction has been published at the time of writing.

### 3.3. Discussion

In study 2, we assessed the analytic reproducibility of key outcomes reported in 35 articles that were deemed to have available and in-principle reusable data. Specifically, we attempted to recover the reported outcomes by repeating the reported analyses upon the shared data. In line with our expectation, we encountered considerable difficulties establishing analytic reproducibility. Prior to requesting assistance from original authors, we achieved a reproducibility success rate of just 31%. This is comparable to the success rates observed in other assessments of analytic reproducibility in political science [19] and economics [20], but is substantially worse than a recent study of clinical RCTs [22]. This lack of analytic reproducibility compromises the credibility of the reported outcomes because they cannot be traced to their source. Furthermore, the utility of any subsequent data reuse is undermined because it is unclear what the results of any novel analyses should be compared with, or whether the outcomes are sufficiently credible to be included in meta-analyses [37].

For all reproducibility issues, we requested, and received, assistance from the original authors. In almost all cases, the responses were prompt, detailed and helpful. Ultimately, 31% of cases were resolved through these discussions, and sometimes the exchange of additional data and/or code. It is encouraging that authors were responsive and took these reproducibility issues seriously. However, relying on extended back-and-forth discussions between data generators and data reusers is clearly not a sustainable model if data sharing and reuse is to become more routine. It is important to note that the sampled articles were all published relatively recently: it seems likely that author responsiveness will decline over time since publication, as e-mail addresses become outdated, careers change, and relevant files are misplaced [16].

In 37% of articles reproducibility issues could not be entirely resolved. Crucially, however, there were no clear indications that original conclusions based on the target outcomes were seriously undermined by the reproducibility issues we encountered. We arrived at this determination through careful consideration of multiple factors, such as the presence/absence of decision errors, the number of target outcomes that could not be reproduced, the type of outcomes that could be reproduced, the difference in magnitude of effect sizes and the predictions of the specific hypothesis under scrutiny. This aspect of our findings necessarily required subjective interpretation and is constrained only to the target outcomes we specifically assessed (which were typically a subset of all outcomes reported in the entire article). For three articles, we were unable to complete aspects of the analysis, and for these cases it is less clear if the original conclusions are affected or not. It is possible that further assessment of the articles would reveal additional problems that would undermine the original conclusions, but this is unknown.

Similarly, it was not always clear whether the non-reproducible target outcomes are actually erroneous (i.e. originally calculated/reported incorrectly) or are accurate and cannot be reproduced (i.e. originally calculated/reported correctly but the analysis pipeline to recreate them cannot be reconstructed). In some cases, we were eventually able to establish a likely cause of the problem: for example when we could not reproduce a single value, but all other outcomes could be reproduced successfully, it seemed fairly certain that the reported value was simply a typographical error (original authors often confirmed this was the most likely cause). However, in many of the non-reproducible cases, the locus of the problem could not be identified, despite extensive exchanges with the original authors.

A common problem we encountered was unclear, incomplete, or incorrect specification of the data analysis pipeline in the original article. *Cognition*'s guidelines specifically state that authors should ‘Provide sufficient details to allow the work to be reproduced by an independent researcher’ [24]. By contrast, many authors appear to have assumed that relevant information was conveyed implicitly or simply did not realize that important details were missing. This could be partly because authors never anticipated someone would try to repeat the original analyses. For example, in one case, the authors had reported participant exclusions in the article, but we were unable to identify who they were referring to in the actual data file. Additionally, authors may not always be aware of the ‘hidden’ defaults employed by statistical software. For example, R defaults to a Welch *t*-test and SPSS reports both the Student *t*-test and the Welch *t*-test [39]. Nevertheless, we also encountered cases where information (e.g. full model specification, data exclusions) should have been reported in the article as it would have been pertinent for reviewers or readers evaluating the article. It may be helpful to provide more specific guidance to authors about the level of detail required to attain analytic reproducibility and enable full evaluation of the study's analytic methods, and reinforce this guidance during the editorial process.

An additional measure to address analysis specification issues would be to require the provision of analysis scripts that describe the pipeline in far greater detail than can be achieved in normal prose. Analysis scripts may contain the actual code used to run the original analysis or syntax output from point-and-click software (e.g. SPSS). If code and syntax are not available, authors could still provide a detailed step-by-step walk-through of the analysis procedure, perhaps accompanied by screenshots. Unfortunately, the findings of study 1 show that overall few authors (18) shared analysis scripts alongside data. Nevertheless, whenever authors did make analysis scripts directly available to us through correspondence, we had the impression that it was much more straightforward to re-implement the analyses and, if necessary, retroactively determine causes of non-reproducibility. Authors can greatly improve the traceability of reported outcomes, and reduce the likelihood of typographical errors, by taking advantage of technologies like R Markdown which interleave analysis code with regular prose to generate reproducible documents [42,43]. These documents can also be shared in online software ‘containers’ that handle software dependency issues and enable reported outcomes to be regenerated with a single click of the mouse [44]. The present manuscript provides a working example (see Data accessibility below).

Policies that mandate sharing of analysis scripts have already been implemented at some journals, for example in economics and political science [19,45,46]. However, it is important to consider that the practical realization of such a policy change may not be straightforward. Whether shared analysis scripts would actually be reusable by independent researchers in practice is unclear. It seems likely that, given the problems we encountered with the reusability of datasets, we would also encounter similar problems with the reusability of analysis scripts. Some journals, such as the *American Journal of Political Science*, have already gone a step further, and employ a third-party organization to check the analytic reproducibility of all articles before publishing them [47]. Under this scheme, authors are not only required to share their analysis scripts, they must also ensure that they are sufficiently well organized and documented such that independent researchers can establish the analytic reproducibility of the reported findings.

It is important to note that study 2 was based on a sample that is likely to provide an optimistic estimate of analytic reproducibility. Firstly, we assessed only a small subset of outcomes, and thus we cannot be certain whether other values reported in the target articles were reproducible. If we had tried to reproduce all analyses in each target article, it seems likely that the number of reproducibility issues would have increased. Secondly, we only attempted to reproduce outcomes related to a substantive finding reported in each article. Given their importance, these are probably the outcomes most carefully checked and verified by the original authors (and/or reviewers). Thirdly, we re-implemented only the most straightforward analyses (ANOVAs, *t*-tests etc.) reported in these articles. It seems likely that more complex analyses would be less straightforward to reproduce because there are inherently more ‘moving parts’ and thus more things that can go wrong.^13^ Fourthly, articles were randomly selected, but from within a corpus of articles that had been submitted to a journal with an especially stringent open data policy, and had already been determined to have shared in-principle reusable data. All else being equal, authors who are willing to share their data, and go to the effort of making it reusable for others, are probably more likely to employ reproducible analysis pipelines. Overall, whether our findings will generalize to other contexts is not straightforward to determine, but it seems likely that they would mostly overestimate reproducibility success. Unfortunately, estimating the prevalence of analytic reproducibility at a larger scale is likely to require substantial resources given the time and effort involved even for the most straightforward analyses examined in this study.

Assessing the implications of our findings is complex and they could be viewed positively or negatively from different perspectives. The fact that almost two-thirds of the cases were eventually reproduced successfully, and none of the original conclusions based on the target outcomes appears to have been seriously undermined, suggests a suboptimal but reasonably healthy situation with regard to the credibility of the original findings. However, the fact that approximately two-thirds of cases were *not* reproducible before author assistance was requested and the time and effort involved to evaluate even relatively straightforward analyses imply a serious deterrent for any routine data reuse.

## 4. General discussion

The credibility and efficiency of the scientific ecosystem is undermined if the research community does not have access to the data that underlie scientific claims [48,49]. Psychological science, in particular, has a poor record on data sharing [8,12,13], and is currently engaged in a field-wide conversation regarding credibility and scientific practices [17]. The introduction of a mandatory open data policy at the journal *Cognition* is an encouraging and progressive response to these problems. Amid a landscape of generally absent or weak data-sharing policies at journals across multiple scientific disciplines, *Cognition*'s policy can be regarded as pioneering [7,15,46].

It is vital that new open science initiatives are iteratively evaluated and improved to maximize their benefit and minimize any negative consequences [50]. In the present investigation, our goal was to examine whether the in-principle benefits of data sharing are actually being realized in practice. Do open data policies actually increase data availability, and, if so, are those shared datasets actually reusable? Most fundamentally, if one has access to the raw data of a published study, can one repeat the reported analyses and obtain the reported outcomes? Such analytic reproducibility is a minimum credibility threshold that all published findings should meet, but it has not been systematically assessed before within a subfield of psychology. Our findings were both encouraging and concerning.

The open data policy was clearly successful at increasing data availability and in-principle reusability, but also fell short of ideal. Many articles in the post-policy period did not report available data, and approximately one-third of the shared data did not appear to be reusable in principle. Open data alone are clearly not enough to achieve the benefits envisaged by proponents of data sharing [2]. For data to have utility, they must be clearly structured and sufficiently well documented. Improved data-sharing guidelines from journals may help to improve this situation, but it is also important that researchers have the necessary skills to implement transparent research practices [36] and are appropriately incentivized [35].

Our reusability assessments only involved relatively brief visual inspection and are therefore likely to underestimate reusability in practice. When we attempted to establish the analytic reproducibility of a subset of articles with in-principle reusable data, we encountered considerable difficulties. Conducting an analytic reproducibility check without an analysis script is rather like assembling flat pack furniture without an instruction booklet: one is given some materials (categorized and labelled with varying degrees of helpfulness), and a diagram of the final product, but is missing the step-by-step instructions required to convert one into the other. In many cases, we found it necessary to call the helpline, and, while the response was helpful, the process became excessively time-consuming and frustrating. However, importantly, there were no clear indications that any original conclusions were seriously undermined by the reproducibility issues we encountered.

The initial non-reproducibility of approximately two-thirds of the assessed cases, and the substantial time and effort expended attempting to establish analytic reproducibility, implies a rather serious deterrent to any scientist considering reusing shared data. Colleagues could be excused if they gave up before spending 25 h on such a task. Neither original authors nor scientists interested in reusing data want to spend their time engaging in extensive back-and-forth exchanges just to establish the analytic reproducibility of published findings. It is therefore in everyone's best interests to ensure that reproducible workflows are adopted during the original study whenever possible [5,36,51].

There is currently a great deal of focus on the relative merits of ‘carrot’ versus ‘stick’ approaches to encourage or mandate transparent and reproducible research practices. Is it preferable, for example, to encourage voluntary transparency by offering authors ‘badges’ to signal their behaviour to the research community [10]? Or should journals be mandating transparency (where possible), as in the present investigation [18]? Carrots and sticks are undoubtedly important instruments of change, and provide an important indication to the community of prevailing cultural norms. But we should not overlook the broader picture. Scientists are only human and inherit all the fallibilities that come with that [52]. From this perspective, it is not surprising that analysis pipelines are peppered with errors, ranging from the mundane to the serious [53–55]. Beyond the carrots and the sticks, it will be highly constructive to turn our attention to the nature of the systems in which scientists operate. By reconfiguring such systems (data analysis pipelines, publishing practices, etc.) in such a manner that *they assume errors are inevitable*, we can start to insert appropriate safeguards to mitigate them. In the present context, a great deal can be learned about reducing errors in analysis pipelines from computer scientists who have developed ‘defensive programming strategies’ to reduce the likelihood of error in computational work [5,51].

Psychological science is currently navigating a period of crisis and opportunity [17,56,57]. Some journals have heeded the growing calls for adoption of transparent and reproducible research practices and introduced new policies to implement change [1,2]. But no new journal policy will be perfect upon first implementation. A new ‘open science frontier’ is blossoming with novel ideas, and initiatives and meta-scientific investigations, such as the present study, are beginning the process of iteratively evaluating and improving proposed reforms [50]. The mandatory open data policy introduced at the journal *Cognition* is a pioneering effort that led to marked increases in data availability and in-principle reusability, but fell short of the optimal. In particular, it is clear that the ideals of data sharing in principle are not yet being realized in practice. Many shared datasets did not appear to be reusable, and we encountered considerable barriers in our efforts to establish analytic reproducibility for a subset of articles, barriers that are likely to be prohibitive for most scientists interested in reusing shared data.

## Supplementary Material

Supplementary materials

## Footnotes

1 The study preregistration stated that there were 609 empirical articles published during this period, but 18 of these papers turned out to be non-empirical when evaluated in more detail by our coders (available here: https://osf.io/rysg8/).

2 Note that by ‘submitted’ we technically mean that the article was formally logged at the journal following author submission. Cognition refers to this as the ‘received date’ in article headers.

3 Note that our preregistration indicated that we would conduct a ‘regression-discontinuity analysis’; however, when time is the variable determining the point of discontinuity, the more commonly used terminology is ‘interrupted time series’, which we employ here. Also note that we did not pre-specify the details of this analysis, such as the exact model specification.

4 We visually inspected the density of DAS inclusion over time and found no evidence that articles were preferentially submitted just before the implementation date.

5 This specification appeared to model the secular trend adequately because the resulting residuals showed no evidence of autocorrelation.

6 We attempted to fit alternative model specifications that would directly model the probability of DAS inclusion (i.e. a linear probability model) or its risk ratio (i.e. a log-linear model), either of which would obviate conversion from the odds ratio to the risk ratio scale. However, the former appeared to fit worse than a logistic regression based on the Akaike information criterion, and the latter failed to converge due to the inclusion of a continuous predictor.

7 Each reusability criterion contained more granular response options (e.g. ‘partly’, ‘some’, ‘unclear’) in addition to binary ‘yes’ or ‘no’ options. As these nuanced responses can be considered a subset of the ‘no’ option, we have combined them with the ‘no’ category for all subsequent analyses. This decision was made *post hoc*, but it allows for more straightforward comparison with the ‘yes’ category. The raw numbers for these more granular responses are as follows: accessibility (‘some’: 0 cases), completeness (‘unclear’: 14 cases; ‘some’: 13 cases), understandability (‘partly’: 41 cases).

8 Note that in some cases (particularly *p*-values and occasionally tests statistics) original outcomes were reported in relation to a boundary rather than their exact value (e.g. *p* < 0.05). As we could not calculate PE in these cases, we simply checked whether the value we obtained in our reanalysis matched the stated boundary relationship. It was necessary to assess 159 values in this manner.

9 We did not encounter any cases where an alpha level other than 0.05 was explicitly specified.

10 Note that in the preregistered study protocol (https://osf.io/q4qy3/) we used the words ‘success’ and ‘failure’ to refer to reproducibility outcomes. We have adjusted the terminology to avoid the potential misinterpretation that a reproducibility problem necessarily compromised the substantive conclusions drawn by the original authors.

11 Note that these percentages do not sum to 100 because of rounding.

12 Note that ideally these issues would have been identified during study 1 article coding. However, such issues were not always readily detectable from a visual survey of the data files and only became apparent when the reanalysis was attempted.

13 Conversely, one could argue that more complex analyses are likely to be implemented with greater care. This may be an interesting issue to explore in future work.

## Data accessibility

The study protocol (hypotheses, methods and analysis plan) was preregistered on 7 March 2017 (https://osf.io/q4qy3/). All deviations from this protocol or additional exploratory analyses are explicitly acknowledged. All data exclusions and measures conducted during this study are reported. All data, materials and analysis scripts related to this study are publicly available at https://osf.io/wn8fd/. To facilitate reproducibility, this manuscript was written by interleaving regular prose and analysis code (https://osf.io/wf3as/) using knitr [42] and papaja [43], and is available in a Code Ocean container (https://doi.org/10.24433/CO.abd8b483-c5e3-4382-a493-1fc5aecb0f1d.v2) which recreates the software environment in which the original analyses were performed [44].
