# Supplementary material for: Data availability, reusability, and analytic reproducibility: evaluating the impact of a mandatory open data policy at the journal Cognition
Source: R Soc Open Sci. 2018 Aug 15;5(8):180448. doi: 10.1098/rsos.180448 (PMC6124055; doi:10.1098/rsos.180448)
Supplement: Supplementary materials [file rsos180448supp1.docx]

# Supplementary Materials

*for*

Hardwicke, T. E., Mathur, M. B., MacDonald, K., Nilsonne, G., Banks, G. C., Kidwell, M. C., Hofelich Mohr, A., Clayton, E., Yoon, E. J., Henry Tessler, M., Lenne, R. L., Altman, S., Long, B., & Frank, M. C. (2018). Data availability, reusability, and analytic reproducibility: Evaluating the impact of a mandatory open data policy at the journal Cognition. *Royal Society Open Science*.

# Supplement A: Cognition’s Data Archiving Policy

Cognition aims to encourage meta-analysis and to facilitate understanding of the data underlying articles published in the journal. Authors of all published papers will therefore be asked to make their raw data publicly available whenever possible.

‘Data’ refers to an electronic file containing non-identified responses that are potentially already coded. Normally, the data would represent an early stage of electronic processing, before individual responses have been aggregated. The data must be in a form that allows all reported statistical analyses to be reproduced while retaining the confidentiality of individual participants. This entails that the data are formatted and documented in a way that makes the structure of the data set readily apparent.

All empirical papers must archive their data upon acceptance in order to be published unless the authors provide a compelling reason why they cannot (e.g., expense, confidentiality). The action editor will be the final arbiter of whether the reason is sufficiently compelling.

Data can be made publically available in different ways. Authors can submit their data as supplementary material (through our online submission system, selecting the submission item as ‘Raw data’) together with their manuscript. Alternatively, there are multiple public repositories that are committed to providing public access into perpetuity: examples are the Open Science Framework, Dataverse, re3data.org and the Databibadd list. Personal websites and most departmental websites do not qualify as repositories because they are potentially transient.

If data are archived in a public repository, a link to the data must be provided under the section heading “Supplementary material” in the manuscript. Note that any publication that reports analyses of or refers to archived data will be expected to cite the original publication in which the data were reported.

For supplementary data submitted together with the final draft of an accepted manuscript, please note: Supplementary files supplied will be published online alongside the electronic version of your article in Elsevier Web products, including ScienceDirect: <http://www.sciencedirect.com>. In order to ensure that your submitted material is directly usable, please provide the data in one of our recommended file formats. Authors should submit the material in electronic format together with the article and supply a concise and descriptive caption for each file. For more detailed instructions please visit our artwork instruction pages at <http://www.elsevier.com/artworkinstructions>.

# Supplement B: Additional results

We encountered a number of different file formats: .xls or .xlsx (Microsoft Excel; *n* = 84), .doc or .docx (Microsoft Word; *n* = 84), .csv (*n* = 45), .pdf (*n* = 23), .txt (*n* = 20), .sav or .sps (SPSS; *n* = 13), .dat (*n* = 6), and .zip (*n* = 3). There were a number of other miscellaneous files that we only encountered once (*n* = 22).

For 114 articles the data files were shared under a Creative Commons “CC-BY” license. 1 article shared data under a custom license. 123 articles did not provide a license with their data.

# Supplement C: Exploratory interrupted time series analysis on data reusability


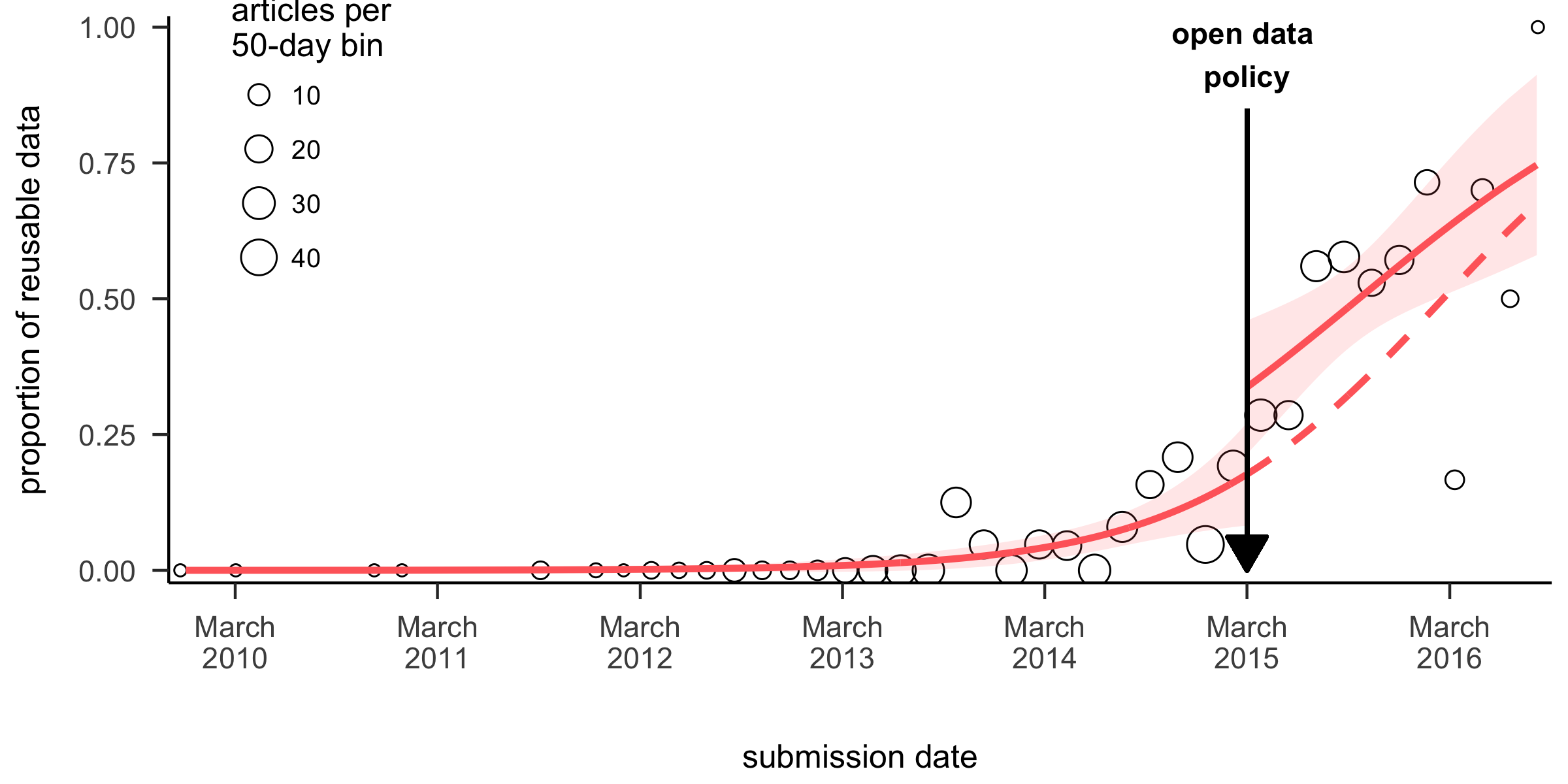


Figure C1. Proportion of articles with in-principle reusable data (available, accessible, complete, and understandable) as a function of submission date across the assessment period. Circles indicate proportions in 50 day bins with circle area representing the total number of articles in each bin. Solid red lines represent predictions of an interrupted time series analysis segemented by pre-policy and post-policy periods. The dashed red line estimates, based on the pre-policy period, the trajectory of in-principle reusability if the policy had no effect. Confidence bands (red) indicate 95% CIs. Note that the small article numbers in the extremes of the graph are due to long submission to publication lag times. Our sample selection was based on publication date, but it is submission date which determines whether an article falls within the pre-policy or post-policy period.

Following on from our analysis of data available statements, we employed an interrupted time series analysis (ITS) to estimate the causal effect of the policy on in-principle reusability (IPR) independent of any contemporary secular trends (see Figure C1). This analysis was not pre-registered. The analysis specifications were identical to those employed for the ITS analysis of data available statements (see main text for details).

During the pre-policy period, we observed a baseline secular trend toward increasing rates of IPR. Specifically, each 50-day passage of time in the pre-period was associated with an estimated 1.19-fold (95% CI [1.09, 1.31], *p* < .001) increase in the probability of IPR. After the open data policy was introduced, there was a substantial “level change” such that data made available immediately after the policy had an estimated 1.89-fold higher probability (95% CI [1.02, 3.05], *p* = .047) of IPR than data made available immediately before the policy.

The trend over time toward increasing rates of IPR did not appear to change substantially in the post-period; the secular trend in the post-period was an estimated 0.96-fold (95% CI [0.83, 1.10], *p* = .568) as large as in the pre-period (a deceleration). As an alternative interpretation, we estimate that 16% (95% CI [0, 40], *p* = .090) of the 50-day secular trend toward increasing IPR in the post-period reflects the baseline secular trend alone, that 77% (95% CI [55, 99], *p* < .001) reflects the effect of the policy alone, and that 7% (95% CI [0, 25], *p* < .001) reflects deceleration in the baseline secular trend due to the policy – that is, the negligible interaction of the policy with the secular trend (Knol & VanderWeele, 2012; Mathur & VanderWeele, 2018; Rothman et al., 1980).

# Supplement D: Exploratory assessment of the ‘population shift’ hypothesis

In order assess the ‘population shift hypothesis’ (see Study One Discussion) we conducted two exploratory analyses examining author retention and journal productivity.

## Autor retention

We examined whether author retention (authors publishing in a given year relative to previous years) at Cognition declined following introduction of the new open data policy. Specifically, we downloaded lists of all authors publishing every year between 2005 and 2017, then computed an ‘author retention index’: the overlap (Sørensen–Dice similarity coefficient; SDC) between each year and the previous three years. The Sørensen–Dice similarity coefficient was calculated as follows:

$$\begin{matrix} \mathrm{SDC} & =\frac{2|X\cap Y|}{|X|+|Y|} \end{matrix}$$

where |X| and |Y| are the numbers of unique authors in the previous three years and a given year respectively. If there was a marked population shift after policy introduction one might expect this to manifest as a decline in the author retention index. Note that some lag time might be expected because of the time taken between article submission and article publication. As shown in Figure D1 below, the author retention index is stable both before and after policy introduction, which is inconsistent with the notion that the post-policy trends in data availability statements and in-principle reusability were substantially influenced by a population shift.


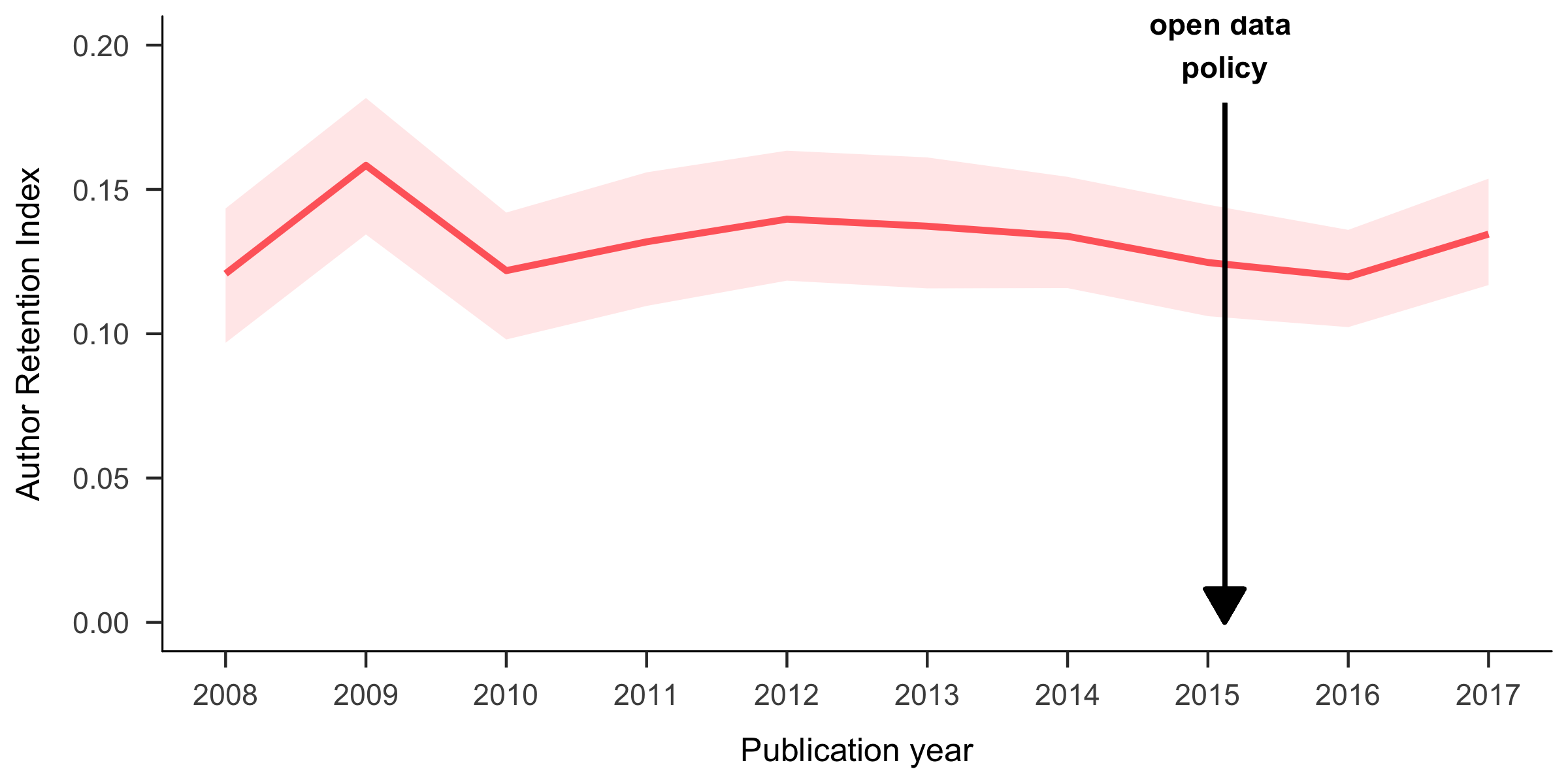


Figure D1. Author retention at Cognition before and after introduction of the open data policy. The Author Retention Index (red line) is the Dice coefficient between a given year and the previous three years. Confidence bands indicate bootstrap 95% CIs.

## Journal productivity

We examined whether journal productivity (number of articles published per year) at Cognition declined following introduction of the new open data policy. We downloaded records for all articles published in Cognition between 2008 and 2017 from The Web of Science using the search terms “SO=Cognition AND PY=(2008-2018)”. If authors were deterred from publishing at Cognition by the open data policy, this could manifest as a decline in the number of publications. Note that some lag time might be expected because of the time taken between article submission and article publication. As shown in Figure D2 below, an increasing trend in the number of publications per years appears to have continued into the post-policy period. This appears to be inconsistent with the notion that large numbers of authors were deterred by the new open data policy. However, it could also be that the number of authors attracted by the policy more than compensated for the number who were deterred.


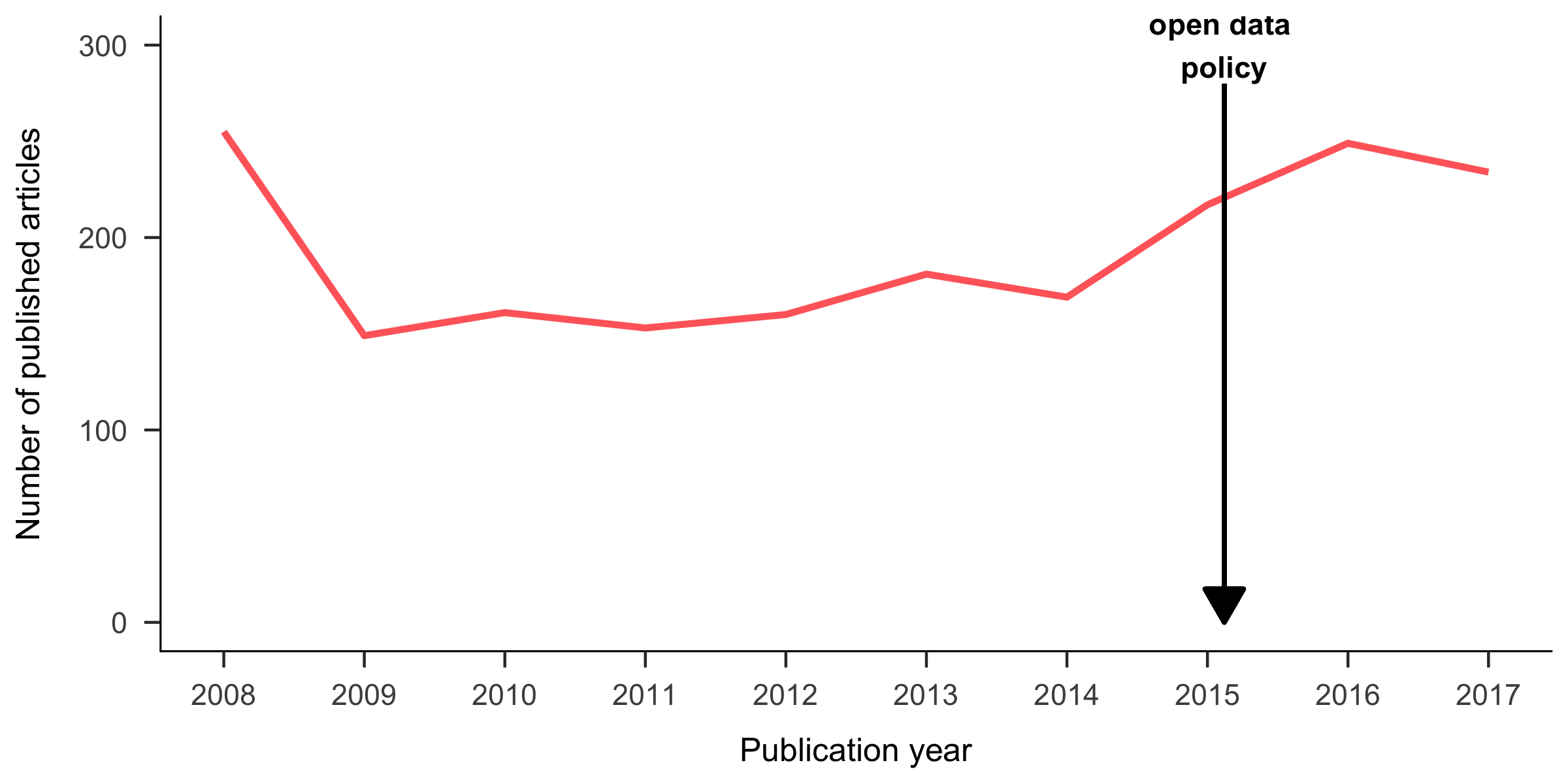


Figure D2. Number of articles published per year at Cognition before and after introduction of the open data policy.

# Supplement E: Reproducibility vignettes

In this section we provide a series of short vignettes briefly summarising the reproducibility checks. Each vignette is accompanied by a link to a reproducibility report written in R Markdown and rendered in HTML, a link to a OSF/Github repository that contains the relevant data and analysis code, and a link to a Code Ocean container which recreates the software environment in which the original analyses were run. The vignettes are presented in order of reproducibility outcome (“Not fully reproducible despite author assistance”, “Reproducible with author assistance”, “Reproducible without author assistance”).

## Vignette 1 (article RPYAj)

Outcome: Not fully reproducible despite author assistance

Substantial implications for the original conclusions: Unlikely

R Markdown report: <http://bit.ly/2C2QD9J>

OSF/Github repository: <https://osf.io/fwtu2/>

Code Ocean reproducible analysis container: <https://doi.org/10.24433/CO.5f3646f8-0b63-46cc-b328-0866c8a92d81>

Description: We encountered nine major errors when reproducing the values reported for a baseline task. The problems affected six standard deviations (SD; reported SD = 47.3 vs. reanalysis SD = 63.8; reported SD = 55.6 vs. reanalysis SD = 63.1; reported SD = 57 vs. reanalysis SD = 103; reported SD = 50.9 vs. reanalysis SD = 66.1; reported SD = 51 vs. reanalysis SD = 58; reported SD = 51.7 vs. reanalysis SD = 90), one mean (reported mean = 448 vs. reanalysis mean = 502), and two F-statistics (reported F = 14.17 vs. reanalysis F = 15.71; reported F = 15.48 vs. reanalysis F = 12.72). We contacted the authors, and they attempted to reproduce the values themselves. They said that they also could not reproduce the values for the baseline task. They were not sure why these problems occurred, but it could be that the data file was corrupted at some stage when manual modifications were made.

Nevertheless, a data file containing the reaction times (RTs) for the main analysis already corrected for baseline was also shared with the article, and the authors said that they are confident these values are accurate (they believe that uncorrupted baseline values were used in the correction process). We were able to reproduce all target outcomes of the main analysis using this already corrected data file.

Although we encountered serious problems reproducing the outcomes of the baseline task, we could successfully reproduce all target outcomes related to the main analysis when using a data set that had already been pre-processed by the original authors. Thus, assuming the pre-processed data set is accurate, it seems unlikely that the reproducibility issues we encountered have substantial implications for the original conclusions.

## Vignette 2 (article TvdWP)

Outcome: Not fully reproducible despite author assistance

Substantial implications for the original conclusions: Unlikely

R Markdown report: <http://bit.ly/2GYgwLG>

OSF/Github repository: <https://osf.io/cfzye/>

Code Ocean reproducible analysis container: <https://doi.org/10.24433/CO.847aff33-8a6f-4860-b86d-f930c997ea8d>

Description: We were initially able to reproduce the descriptive statistics but could not reproduce the reported 95% confidence intervals (CIs) when we implemented the formula provided in a footnote. We contacted the original authors and they sent their analysis code. This enabled us to spot that their implementation of the formula contained an error, specifically a parentheses error that resulted in an incorrect calculation of the pooled standard deviation. The original authors also identified the same problem independently after checking their own code. When the problem was corrected it increased the width of the confidence intervals relative to those reported. With the provision of code, we could reproduce all of the CIs except for one upper bound (reported CI = -0.1 vs. reanalysis CI = -9.2). The change in the width of the CIs might change the appraisal of estimate uncertainty, but most target outcomes were reproduced successfully, and there were no ‘decision errors’, thus it seems unlikely that these reproducibility issues have substantial implications for the original conclusions.

## Vignette 3 (article KIUKz)

Outcome: Not fully reproducible despite author assistance

Substantial implications for the original conclusions: Unlikely

R Markdown report: <http://bit.ly/2ER14k1>

OSF/Github repository: <https://osf.io/4h997/>

Code Ocean reproducible analysis container: <https://doi.org/10.24433/CO.1d672081-fbdc-4ffd-867a-9bcefdc09385>

Description: We initially could not reproduce the participant-level descriptive statistics using the provided trial-level data. We contacted an original author and received a prompt reply which clarified that we were implementing the analysis incorrectly. The precise analyses were not entirely detailed in the paper, but it is possible that an individual with more experience of this particular theoretical framework (signal detection theory) would have understood the procedures as standard practice.

With assistance, we could successfully reproduce the descriptive statistics, however we then ran into further problems with the inferential statistics. Most inferential outcomes were reproduced successfully, but there were major errors for two F-statistics (reported F = 60.2 vs. reanalysis F = 50.9; reported F = 151.4 vs. reanalysis F = 109.8) and three degrees of freedom (reported DF = 22 vs. reanalysis DF = 46; reported DF = 22 vs. reanalysis DF = 46; reported DF = 22 vs. reanalysis DF = 46). We followed up with the authors a second time to address these issues but received no response to our initial e-mail or a 2 week follow-up reminder. As the issues cannot be resolved, this reproducibility check has to be classified as not fully reproducible despite author assistance. Nevertheless, considering the magnitude of the errors, and the absence of decision errors, it seems unlikely that these reproducibility issues have substantial implications for the original conclusions.

## Vignette 4 (article UYYED)

Outcome: Not fully reproducible despite author assistance

Substantial implications for the original conclusions: Unlikely

R Markdown report: <http://bit.ly/2EtGzf8>

OSF/Github repository: <https://osf.io/dn7a6/>

Code Ocean reproducible analysis container: <https://doi.org/10.24433/CO.b86d511c-56e7-4383-94f3-8dc4923c5717>

Description: We initially encountered problems reproducing many values in this article. Investigating the source of the problem, we came across inconsistencies within the data file itself. Some data also appeared to be missing. We contacted the authors for clarification and they confirmed that the data file contained errors and missing values. They suggested the problems had been introduced during manual editing of the data file in order to make it more user-friendly. The authors provided a new data file, but we still encountered reproducibility problems when we re-ran our analysis. The authors provided some additional information about data aggregation which helped to resolve some reproducibility issues. However, when they checked their own analyses, the authors also found that some of the values reported in the paper were based on a previous version of the data set that had not been updated.

Ultimately there were five major errors remaining (reported standard error [SE] = 0.04 vs. reanalysis SE = 0.05; reported p = 0.0014 vs. reanalysis p = 0.0028; reported mean = 0.06 vs. reanalysis mean = 0.05; reported SE = 0.03 vs. reanalysis SE = 0.04; reported p = 0.05 vs. reanalysis p = 0.04). Although there were several errors, considering their magnitude and the absence of ‘decision errors’, it seems unlikely that these reproducibility issues have substantial implications for the original conclusions. The authors have now published a correction in Cognition.

## Vignette 5 (article OvGDB)

Outcome: Not fully reproducible despite author assistance

Substantial implications for the original conclusions: Unlikely

R Markdown report: <http://bit.ly/2HXqBc9>

OSF/Github repository: <https://osf.io/6zbfm/>

Code Ocean reproducible analysis container: <https://doi.org/10.24433/CO.8b51826d-e18e-4bd2-8817-2802975f82e8>

Description: We initially encountered two reproducibility issues, one for a proportion and one for an effect size. We contacted the authors for clarification. The proportion was correctly reported but the specification of the analysis was ambiguous. With author clarification we were able to reproduce this value. The effect size (reported d = 0.65 vs. reanalysis d = 0.23) was incorrectly reported but the source of the error is unknown to us or the authors. Because all other target outcomes were reproducible and there were no ‘decision errors’, it seems unlikely that the reproducibility issues have substantial implications for the original conclusions.

## Vignette 6 (article DvcDF)

Outcome: Not fully reproducible despite author assistance

Substantial implications for the original conclusions: Unlikely

R Markdown report: <http://bit.ly/2EafgmI>

OSF/Github repository: <https://osf.io/9dgbh/>

Code Ocean reproducible analysis container: <https://doi.org/10.24433/CO.70bca8a5-353d-4751-9e83-df87c826dc8c>

Description: We initially encountered a large number reproducibility issues in the descriptive and inferential statistics. We contacted the original authors for clarification and they said that the issues arose because the provided data file contained rounded values rather than unrounded values. The authors sent us a new data file and we re-ran our analyses. Many issues were resolved, but several still remained. In further correspondence, the original authors reported that they had re-run their own analyses (they also sent us the analysis script). They informed us that they could also not reproduce some of the reported values, although could approximate most of them: they believe that most of the mismatches are rounding errors or typos. They also identified one data entry error, and one aspect of the analysis (model specification) that had not been reported in the original article.

Ultimately, a number of discrepancies remained (six major errors) between the output of our analyses and the authors’ analyses (reported t = 0.66 vs. reanalysis t = 0.85; reported p = 0.51 vs. reanalysis p = 0.4; reported d = 0.17 vs. reanalysis d = 0.22; reported p = 0.26 vs. reanalysis p = 0.2, reported d = 0.29 vs. reanalysis d = 0.33; reported variance = 26127 vs. reanalysis variance = 21928). Although there were several errors, considering their magnitude and the absence of ‘decision errors’, it seems unlikely that these reproducibility issues have substantial implications for the original conclusions.

## Vignette 7 (article jjmld)

Outcome: Not fully reproducible despite author assistance

Substantial implications for the original conclusions: Unclear

R Markdown report: <http://bit.ly/2E6ky2D>

OSF/Github repository: <https://osf.io/6bjn9/>

Code Ocean reproducible analysis container: <https://doi.org/10.24433/CO.44cf523c-4d3f-4fcb-97c6-aca87b976921>

Description: Initially we were unable to proceed with the analysis because necessary information was not provided in the data file. Specifically, responses were not identified as correct or incorrect, and we needed this information in order to calculate the response times, which were based only on trials with correct responses. Additionally, one dependent variable was not present in the data file. We contacted the original authors and they sent new data files containing the missing information. However, they also said that they had run their own analysis and could not reproduce some of the reported values (they did not specify which ones).

We continued to attempt the reanalysis with the new data files in order to estimate the number of reproducibility errors. We encountered 13 major numerical errors and one insufficient information error. Specifically, we could not reproduce the number of participants who did not respond on a task (reported n = 4 vs. reanalysis n = 5), three standard deviations (SDs; reported SD = 284 vs. obtained SD = 338; reported SD = 432 vs. reanalysis SD = 528; reported SD = 524 vs. reanalysis SD = 584), five p-values (reported p = 0.01 vs. reanalysis p = 0.009; reported p = 0.659 vs. reanalysis p = 0.588; reported p = 0.175 vs. reanalysis p = 0.316; reported p = 0.623 vs. reanalysis p = 0.902; reported p = 0.002 vs. reanalysis p = 0.003), one partial-eta squared (PES; reported PES = 0.01 vs. reanalysis PES = 0.02), and three F-values (reported F = 0.2 vs. reanalysis F = 0.31; reported F = 13.98 vs. reanalysis F = 12.31; reported F = 0.08 vs. reanalysis F = 0.09).

Although there were numerous errors, they do not appear to be of substantial magnitude, and there were no decision errors. However, because we could not complete part of the analysis, it is unclear whether the reproducibility issues have substantial implications for the original conclusions.

## Vignette 8 (article COGGV)

Outcome: Not fully reproducible despite author assistance

Substantial implications for the original conclusions: Unlikely

R Markdown report: <http://bit.ly/2GYG776>

OSF/Github repository: <https://osf.io/u8uqv/>

Code Ocean reproducible analysis container: <https://doi.org/10.24433/CO.d611b7a7-8a18-4cb3-8ce5-171c11b2793e>

Description: We encountered five major reproducibility errors affecting the inferential statistics (reported t = 2.87 vs. reanalysis t = 3.63; reported partial eta-squared [PES] = 0.28 vs reanalysis PES = 0.39; reported t = 0.13 vs. reanalysis t = 0.02; reported PES = 0.001 vs. reanalysis PES = 0.000021; reported p = 0.89 vs. reanalysis p = 0.98).

The locus of the reproducibility issues remains unclear. We suspected it could be related to the implementation of the exclusion criteria earlier in the analysis pipeline as there was some ambiguity about this in the original article. We attempted to resolve these issues by contacting the original author and received some responses. However, the issues were not addressed in initial correspondence and we did not receive replies to two additional follow-up e-mails. The target outcomes were therefore not fully reproducible despite author assistance. Considering the magnitude of the errors and the absence of ‘decision errors’, it seems unlikely that these reproducibility issues have substantial implications for the original conclusions.

## Vignette 9 (article DRaKS)

Outcome: Not fully reproducible despite author assistance

Substantial implications for the original conclusions: Unclear

R Markdown report: <http://bit.ly/2ENzMuC>

OSF/Github repository: <https://osf.io/g5y6a/>

Code Ocean reproducible analysis container: <https://doi.org/10.24433/CO.fd1d01e0-3aea-487a-a053-0ab740ed6824>

Description: We were able to reproduce some target outcomes, but we encountered 5 major numerical differences for a set of p-values, and there was some data missing which prevented us from running part of the analyses. The p-values differed in magnitude, but were not ‘decision errors’ (reported p = 2.89e-15 vs. reanalysis p = 3.55e-15; reported p = 9.08e-14 vs. reanalysis p = 1.776e-13; reported p = 2.89e-15 vs reanalysis p = 3.55e-15; reported p = 9.08e-14 vs. reanalysis p = 1.776e-13; reported p = 3.24e-8 vs. reanalysis p = 7.6e-9).

The original authors suggested that if we excluded a participant from these analyses (which was not specified in the original article) it would resolve the p-value issue. However, we were still unable to reproduce the target values after implementing the exclusion. Regarding the missing data, the original authors were unable to locate this data and believe it is lost. Because we were unable to conduct part of the analysis, it is unclear if the reproducibility issues have substantial implications for the original conclusions.

## Vignette 10 (article rggwx)

Outcome: Not fully reproducible despite author assistance

Substantial implications for the original conclusions: Unlikely

R Markdown report: <http://bit.ly/2CoTnCT>

OSF/Github repository: <https://osf.io/ej3b9/>

Code Ocean reproducible analysis container: <https://doi.org/10.24433/CO.380040f8-9d10-4811-8fc9-2bb1e3a52b0b>

Description: We initially encountered a problem implementing the exclusion criteria: The exclusion rate we obtained did not match the one reported (reported exclusion rate = 1.2% vs. reanalysis exclusion rate = 1.35%). After corresponding with the original author, it transpired that the reported exclusion rate was incorrect, and in their own reanalysis the author obtained the same value that we did.

Subsequently, we encountered numerous errors reproducing the descriptive statistics. The authors informed us that there was an additional aggregation step that had not been reported in the article. When we implemented this step, we were able to reproduce most values. However, we were still unable to reproduce four standard deviations (reported SD = 169.6 vs. reanalysis SD = 196.6; reported SD = 9.6 vs. reanalysis SD = 12.1; reported SD = 7.2 vs. reanalysis SD = 29.6; reported SD = 7.1 vs. reanalysis SD = 29.1). The authors found that they could also not reproduce these values in their own reanalysis, and their outcomes concurred with our own. They suggested that the errors may have arisen when copying from the analysis output to the manuscript. Considering the magnitude of the errors, and because all other target outcomes were reproducible, it seems unlikely that this reproducibility issue has substantial implications for the original conclusions.

## Vignette 11 (article IBRbN)

Outcome: Not fully reproducible despite author assistance

Substantial implications for the original conclusions: Unlikely

R Markdown report: <http://bit.ly/2F9sqUQ>

OSF/Github repository: <https://osf.io/qsf89/>

Code Ocean reproducible analysis container: <https://doi.org/10.24433/CO.f28dc2d0-7b7b-43ea-84a2-6017165716b7>

Description: We were able to successfully reproduce most target outcomes, however, there was a mismatch for one degrees of freedom (DF) value reported in an ANOVA (reported DF = 2 vs. reanalysis DF = 1). We contacted the authors and they told us that this must have been a typo. Considering the magnitude of the error, and because all other target outcomes were reproducible, it seems unlikely that this reproducibility issue has substantial implications for the original conclusions.

## Vignette 12 (article IeIFy)

Outcome: Not fully reproducible despite author assistance

Substantial implications for the original conclusions: Unlikely

R Markdown report: <http://bit.ly/2BKDZke>

OSF/Github repository: <https://osf.io/s2xbb/>

Code Ocean reproducible analysis container: <https://doi.org/10.24433/CO.213018ba-0a5d-409b-99c2-031fd1705165>

Description: We initially found that the inferential statistics matched those reported in the paper, but there were inconsistencies with some of the descriptive statistics. Specifically, there were four major numerical errors affecting two mean difference scores (MDSs; reported MDS = 14.27 vs. reanalysis MDS = 23.4; reported MDS = 5.93 vs. reanalysis MDS = 11.56) and two standard deviations (SDs; reported SD = 19.49 vs. reanalysis SD = 38.13; reported SD = 3.54 vs. reanalysis SD = 4.47).

Fortunately, the authors had shared their analysis code in the supplementary material, and we found that the values reported in this file matched ours, and not those reported in the paper. We contacted the original authors for clarification and they confirmed that these specific values reported in the article are incorrect. The values we obtained, and those reported in the supplementary materials, are the correct values. The authors suggested that these errors arose because they did not update the manuscript after updating some aspects of the analysis. Because most other descriptive target outcomes, and all inferential target outcomes were reproducible, it seems unlikely that the reproducibility issues have substantial implications for the original conclusions.

## Vignette 13 (article bPJii)

Outcome: Not fully reproducible despite author assistance

Substantial implications for the original conclusions: Unclear

R Markdown report: <http://bit.ly/2FSkYhm>

OSF/Github repository: <https://osf.io/y4qk5/>

Code Ocean reproducible analysis container: <https://doi.org/10.24433/CO.3c0e0ca6-6078-4022-9e12-e5efd8fcd0e9>

Description: We initially encountered considerable problems implementing the pre-processing steps necessary to get the raw data ready for analysis. There did not seem to be sufficient information provided in the article to implement these steps. We contacted the authors for assistance and they promptly replied with some instructions, Matlab code, and already pre-processed data. We still could not get the preprocessing to work so instead we attempt to use the provided Matlab code and already pre-processed data to replicate the target outcomes.

Overall, the Matlab code and additional data helped considerably, and we were able to approximate some of the target outcomes. However, there were still four major reproducibility errors (reported p = 0.51 vs reanalysis p = 0.58; reported r = -0.18 vs reanalysis r = -0.15; reported p = 0.31 vs reanalysis p = 0.38; reported r =-0.27 vs reanalysis r = -0.23). Additionally, some aspects of the analysis were not addressed in the provided code, such as the removal of one stimuli type and the correlations between pupil and fixation. We could not identify the stimuli type that needed to be excluded, and recorded an insufficient information error.

Ultimately, despite helpful assistance from the original authors, some target outcomes were not reproducible. We did request additional assistance from the original authors but they did not respond to this last query. Because we were unable to conduct some aspects of the analysis, we cannot be certain whether the reproducibility issues have substantial implications for the original conclusions.

## Vignette 14 (article UAIUi)

Outcome: Reproducible with author assistance

Substantial implications for the original conclusions: No

R Markdown report: <http://bit.ly/2C2D3TT>

OSF/Github repository: <https://osf.io/cd867/>

Code Ocean reproducible analysis container: <https://doi.org/10.24433/CO.b65ec9d9-2229-44c8-91fb-a80ca69b8e27>

Description: We initially encountered problems reproducing a standard deviation value for one aspect of the analyses. We contacted the authors for clarification and they provided more detail about the approach they had used to aggregate the data. This was not the approach we had assumed from reading the paper. Nevertheless, with this additional information, we were successfully able to reproduce all target outcomes.

## Vignette 15 (article crIXN)

Outcome: Reproducible with author assistance

Substantial implications for the original conclusions: No

R Markdown report: <http://bit.ly/2GZrBw8>

OSF/Github repository: <https://osf.io/q49rp/>

Code Ocean reproducible analysis container: <https://doi.org/10.24433/CO.872ca06d-e45a-4ab9-b333-f5fa5d65701b>

Description: We could initially reproduce most target outcomes with some exceptions. Firstly, we did not have sufficient information about the specification of Bayes Factor calculations, Secondly, we could not reproduce some standard errors. We contacted the authors for clarification and they provided more detailed specification of the Bayes Factor analysis. Additionally, they informed us that the reported standard errors had been adjusted to take into account within-subject variance (this was not mentioned in the article), based on an algorithm introduced by Cousineau (2005). This requires norming the data first. After taking following these additional steps, we could successfully reproduce all target outcomes.

## Vignette 16 (article Tbkij)

Outcome: Reproducible with author assistance

Substantial implications for the original conclusions: No

R Markdown report: <http://bit.ly/2EP0wuL>

OSF/Github repository: <https://osf.io/8vvvj/>

Code Ocean reproducible analysis container: <https://doi.org/10.24433/CO.7464be3a-4795-44fe-aaf6-a899ff528011>

Description: There was initially insufficient information to continue with the analysis. Specifically, the original article referred to two participant exclusions, but it was unclear which participants in the data file were excluded. Additionally, two inferential tests were not explicitly identified (only p-values were reported). After discussion with an original author, we were able to identify the excluded participants and the inferential tests employed. We were then able to successfully reproduce all target outcomes.

## Vignette 17 (article gIjYG)

Outcome: Reproducible with author assistance

Substantial implications for the original conclusions: No

R Markdown report: <http://bit.ly/2nRTu09>

OSF/Github repository: <https://osf.io/8b2uz/>

Code Ocean reproducible analysis container: <https://doi.org/10.24433/CO.b2a8ce0d-01dc-4e79-8d61-68667bb65030>

Description: We were initially able to reproduce some of the descriptive statistics but could not reproduce the standard errors. We also did not attempt the ANOVA as it appeared that a correction had been applied and the correction was not identified. We contacted the original authors for clarification. Regarding the standard errors, the authors informed us they calculated “within-subject standard errors” using the Cousineau-Morey method (this was not reported in the original article). They provided the R function used to implement this method, and we were able to reproduce the values successfully with this. Regarding the ANOVA, the authors informed us that a Greenhouse-Geisser correction had been employed (this was not reported in the original article) and provided the specific R code they had used. When we tried to implement the ANOVA using this R function, we still encountered two major numerical errors. The authors then informed us that some of the data had actually been excluded prior to running the main analysis (this was not reported in the original article). The authors also provided code to implement this exclusion and run the ANVOA. With some minor edits, we got this to work and were able to reproduce the target outcomes.

## Vignette 18 (article DFDwT)

Outcome: Reproducible with author assistance

Substantial implications for the original conclusions: No

R Markdown report: <http://bit.ly/2E7GCOB>

OSF/Github repository: <https://osf.io/hx743/>

Code Ocean reproducible analysis container: <https://doi.org/10.24433/CO.3571d425-86dc-45d4-a8c6-f19b89648542>

Description: We initially encountered some difficulties reproducing some standard deviations. However, after discussing the issue with the authors, we discovered that we were aggregating the data differently. When we followed the author’s specification, we could reproduce all target values successfully.

## Vignette 19 (article JcuWB)

Outcome: Reproducible with author assistance

Substantial implications for the original conclusions: No

R Markdown report: <http://bit.ly/2EaM52U>

OSF/Github repository: <https://osf.io/24gnt/>

Code Ocean reproducible analysis container: <https://doi.org/10.24433/CO.a936e462-2a26-4773-88e0-78a6990f2777>

Description: We initially ran into difficulties with this reproducibility check. Specifically, we were able to reproduce the output of the ANCOVA and t-tests fairly closely, but many of the means and confidence intervals were not matching up. The original authors informed us that these values were in fact marginal means rather than sample means (this was not reported in the article). We attempted to calculate the marginal means, and came much closer, but still could not reproduce some values. The authors then provided SPSS syntax which we successfully used to reproduce the values reported in the article.

Provision of the SPSS syntax and corresponding data file also enabled us to solve a secondary issue. We noticed that our normalized age values did not match up exactly with those in the SPSS file. Eventually, we identified the source of the problem - in the original data file, the age for participant 103 is given as “4y8m” i.e, 56 months. However, in the SPSS data file provide to us directly by the original authors, the age for this participant is given as 57 months. When we changed the age to 57, the normalised values matched up. It is not clear why the values in the two data files are different, but it does not seem to have serious repercussions for the substantive conclusions of the original article.

## Vignette 20 (article NoMcC)

Outcome: Reproducible with author assistance

Substantial implications for the original conclusions: No

R Markdown report: <http://bit.ly/2EMVf6Q>

OSF/Github repository: <https://osf.io/2fqbj/>

Code Ocean reproducible analysis container: <https://doi.org/10.24433/CO.03419521-6398-4fc6-b2f6-ef6294c55732>

Description: We were initially able to reproduce the descriptive statistics but had considerable problems reproducing the outcomes of a linear mixed model analysis. We contacted the original authors and they provided the SPSS syntax they had used to calculate the model. Using the more detailed specification provided in this syntax, we were able to reproduce the target outcomes

## Vignette 21 (article UlhiU)

Outcome: Reproducible with author assistance

Substantial implications for the original conclusions: No

R Markdown report: <http://bit.ly/2nKw8KR>

OSF/Github repository: <https://osf.io/ue5k6/>

Code Ocean reproducible analysis container: <https://doi.org/10.24433/CO.af3ad853-6de2-47be-826a-0fc9b5a5f499>

Description: We initially encountered problems reproduce some of the descriptive statistics, although we could successfully reproduce the inferential statistics. Additional, some data was missing and we could not attempt one aspect of the analysis. We contacted the original authors and they provided the missing data and additional information about how the data had been filtered before calculating the descriptive statistics. With the original authors’ help, we were able to reproduce all of the target outcomes.

## Vignette 22 (article Wzqlp)

Outcome: Reproducible with author assistance

Substantial implications for the original conclusions: No

R Markdown report: <http://bit.ly/2GYR5td>

OSF/Github repository: <https://osf.io/ycmxj/>

Code Ocean reproducible analysis container: <https://doi.org/10.24433/CO.16a3f463-b86a-4cea-a359-55e8fd207090>

Description: We initially reproduced the descriptive statistics successfully but had trouble reproduce a t-value and couldn’t attempt part of the analysis because of insufficient information about one type of inferential test used. We contacted the original authors and they clarified the nature of the statistical tests. They also attempted to reproduce the problematic values in their own re-analysis and discovered that the data set shared alongside the article contained rounded values. The outcomes could only be reproduced when the unrounded values were used. The authors sent us the unrounded data and we were able to reproduce all target outcomes.

## Vignette 23 (article AgnZI)

Outcome: Reproducible with author assistance

Substantial implications for the original conclusions: No

R Markdown report: <http://bit.ly/2siTwDX>

OSF/Github repository: <https://osf.io/xtf7u/>

Code Ocean reproducible analysis container: <https://doi.org/10.24433/CO.ede8aa25-191b-40cb-b6ac-8f875378a6f9>

Description: Initially we were able to reproduce all but one value. The article stated that “thirteen out of 15” participants in one group responded correctly, however according to the data file 14 out of 15 responded correctly. The original authors clarified that there was an error in the data file itself - one participant’s response should have been labelled ‘incorrect’ rather than ‘correct’. After making this change, we were able to reproduce all outcomes successfully.

## Vignette 24 (article Xvfpm)

Outcome: Reproducible with author assistance

Substantial implications for the original conclusions: No

R Markdown report: <http://bit.ly/2EsxjYW>

OSF/Github repository: <https://osf.io/dpxn3/>

Code Ocean reproducible analysis container: <https://doi.org/10.24433/CO.ff929333-fe92-419b-89be-679a261000e1>

Description: We were initially unable to reproduce some ANOVA outcomes, and could not proceed with some aspects of the analysis due to insufficient information regarding standardized vs. unstandardized regression coefficients. We contacted the original authors and they provided additional information and R code that enable us to reproduce the reported outcomes. They specific problems were that a Greenhouse-Geisser correction had been applied to the ANOVA outcomes and was not reported in the article, and the specification of standardized vs. unstandardized coefficients was not clearly outlined.

## Vignette 25 (article APNGu)

Outcome: Reproducible without author assistance

Substantial implications for the original conclusions: No

R Markdown report: <http://bit.ly/2C6hGBi>

OSF/Github repository: <https://osf.io/ve5ck/>

Code Ocean reproducible analysis container: <https://doi.org/10.24433/CO.55ee61de-d25f-496b-9824-3db497a2a7bc>

Description: We were able to successfully reproduce all target outcomes. It was not necessary to request assistance from the original authors.

## Vignette 26 (article MbLKW)

Outcome: Reproducible without author assistance

Substantial implications for the original conclusions: No

R Markdown report: <http://bit.ly/2nPdfWg>

OSF/Github repository: <https://osf.io/qb2gs/>

Code Ocean reproducible analysis container: <https://doi.org/10.24433/CO.dd07be20-ea23-4de2-9ec8-5686044227d1>

Description: We were able to successfully reproduce all target outcomes. It was not necessary to request assistance from the original authors.

## Vignette 27 (article hJiYk)

Outcome: Reproducible without author assistance

Substantial implications for the original conclusions: No

R Markdown report: <http://bit.ly/2FTwrK0>

OSF/Github repository: <https://osf.io/jnvxv/>

Code Ocean reproducible analysis container: <https://doi.org/10.24433/CO.e3723378-ccf3-4314-a410-23f161942774>

Description: We were able to successfully reproduce all target outcomes. It was not necessary to request assistance from the original authors.

## Vignette 28 (article ILpNO)

Outcome: Reproducible without author assistance

Substantial implications for the original conclusions: No

R Markdown report: <http://bit.ly/2FS1W79>

OSF/Github repository: <https://osf.io/cm7qv/>

Code Ocean reproducible analysis container: <https://doi.org/10.24433/CO.7f90eea6-34f9-4197-87a6-aa9860ef24f7>

Description: We were able to successfully reproduce all target outcomes. It was not necessary to request assistance from the original authors.

## Vignette 29 (article sDcDq)

Outcome: Reproducible without author assistance

Substantial implications for the original conclusions: No

R Markdown report: <http://bit.ly/2E8Klv7>

OSF/Github repository: <https://osf.io/59u7f/>

Code Ocean reproducible analysis container: <https://doi.org/10.24433/CO.8a96b3be-1dc6-4576-94dd-500659ae329d>

Description: We were able to successfully reproduce all target outcomes. It was not necessary to request assistance from the original authors.

## Vignette 30 (article htkUz)

Outcome: Reproducible without author assistance

Substantial implications for the original conclusions: No

R Markdown report: <http://bit.ly/2EsXHBY>

OSF/Github repository: <https://osf.io/6ymh2/>

Code Ocean reproducible analysis container: <https://doi.org/10.24433/CO.478b0a75-c42f-4322-baba-d71db0fc65fd>

Description: We were able to successfully reproduce all target outcomes. It was not necessary to request assistance from the original authors.

## Vignette 31 (article leydV)

Outcome: Reproducible without author assistance

Substantial implications for the original conclusions: No

R Markdown report: <http://bit.ly/2nSiKDx>

OSF/Github repository: <https://osf.io/vf37r/>

Code Ocean reproducible analysis container: <https://doi.org/10.24433/CO.6e72ad4b-b1e0-4c85-a61c-64abd44a985c>

Description: We were able to successfully reproduce all target outcomes. It was not necessary to request assistance from the original authors.

## Vignette 32 (article LcquD)

Outcome: Reproducible without author assistance

Substantial implications for the original conclusions: No

R Markdown report: <http://bit.ly/2BhztZt>

OSF/Github repository: <https://osf.io/mcq6g/>

Code Ocean reproducible analysis container: <https://doi.org/10.24433/CO.c5502e87-0ec0-442c-8256-3306afb1bb64>

Description: We were able to successfully reproduce all target outcomes. It was not necessary to request assistance from the original authors.

## Vignette 33 (article jCSIW)

Outcome: Reproducible without author assistance

Substantial implications for the original conclusions: No

R Markdown report: <http://bit.ly/2nLI6Ut>

OSF/Github repository: <https://osf.io/bn6vf/>

Code Ocean reproducible analysis container: <https://doi.org/10.24433/CO.05b37042-e98b-4e98-9497-1554f0d1f1db>

Description: We were able to successfully reproduce all target outcomes. It was not necessary to request assistance from the original authors.

## Vignette 34 (article ENIfE)

Outcome: Reproducible without author assistance

Substantial implications for the original conclusions: No

R Markdown report: <http://bit.ly/2BgRkQ3>

OSF/Github repository: <https://osf.io/r6z3h/>

Code Ocean reproducible analysis container: <https://doi.org/10.24433/CO.80388ffb-3fec-460a-8efb-6728e850bac9>

Description: We were able to successfully reproduce all target outcomes. It was not necessary to request assistance from the original authors.

## Vignette 35 (article bGRfF)

Outcome: Reproducible without author assistance

Substantial implications for the original conclusions: No

R Markdown report: <http://bit.ly/2EMgO7y>

OSF/Github repository: <https://osf.io/amhpv/>

Code Ocean reproducible analysis container: <https://doi.org/10.24433/CO.b10c35ea-d775-4c0a-8a66-7794e94b7304>

Description: We were able to successfully reproduce all target outcomes. It was not necessary to request assistance from the original authors.
